# Tri(boryl)alkanes and Tri(boryl)alkenes: The Versatile Reagents

**DOI:** 10.3390/molecules25071758

**Published:** 2020-04-10

**Authors:** Oriol Salvadó, Elena Fernández

**Affiliations:** Department Química Física i Inorgànica, University Rovira i Virgili, 43007 Tarragona, Spain; oriol.salvado@urv.cat

**Keywords:** 1,1,1-tri(boryl)alkanes, 1,2,3-tri(boryl)alkanes, 1,1,2-tri(boryl)alkanes, 1,1,2-tri(boryl)alkenes, synthetic approaches, synthetic applications

## Abstract

The interest of organoboron chemistry in organic synthesis is growing, together with the development of new and versatile polyborated reagents. Here, the preparation of 1,1,1-tri(boryl)alkanes, 1,2,3-tri(boryl)alkanes, 1,1,2-tri(boryl)alkanes, as well as 1,1,2-tri(boryl)alkenes as suitable and accessible polyborated systems is demonstrated as being easily applied in the construction of new carbon-carbon and carbon-heteroatom bonds. Synthetic procedures and limitations have been collected to demonstrate the powerful strategies to construct selective molecules, taking advantages of the easy transformation of carbon-boron bond in multiple functionalities, under the total control of chemo- and stereoselectivity.

## 1. Introduction

The possibility of preparing a compound that contains three C-B bonds within its formula enhances the power towards polyfunctionalization strategies in a sequential manner. The fact that the three boryl units can be bonded to the same C or different carbons of the same single compound opens a large number of possibilities to construct a new functional product, especially from the perspective that the presence of more than one boryl group contributes to the stabilization of the intermediate carbanions formed, facilitating the transformation of the C-B bond, even under room temperature conditions. Both tri(boryl)alkanes and tri(boryl)alkenes preserve the stereoselectivity along the transformations and the current approaches to functionalize chemoselectively one boryl unit versus the other two, which nowadays open an unlimited opportunity to stimulate the creativity of synthetic chemists towards accessible protocols for useful applications.

## 2. 1,1,1-Tri(boryl)alkanes

### 2.1. Synthetic Approaches

**Scheme 1:**
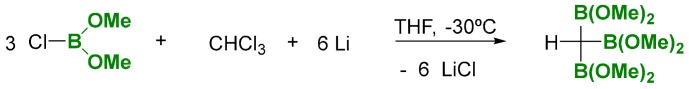
Synthesis of H(B(OMe)_2_)_3._

Triborylmethane compounds have originally been prepared by Matteson and co-workers, developing a reliable synthetic procedure by mixing dimethoxyboron chloride, Cl-B(OMe)_2_, with CHCl_3_ and Li (Scheme 1) [1,2]. By using the appropriate chlorinated reagents, related triboryl compounds can be easily prepared, giving access to a new generation of organopolyborated species. 

**Scheme 2:**
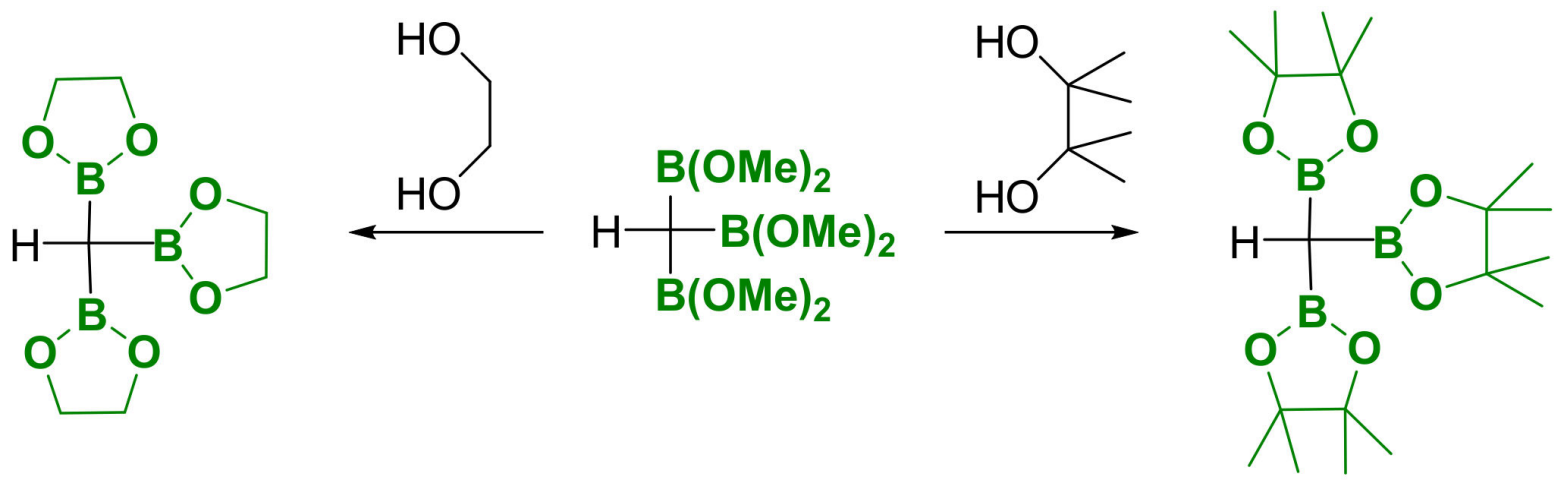
Synthesis of H(Bpin)_3_ from H(B(OMe)_2_)_3._

The lability of dimethoxyboryl moieties contributes to several difficulties in isolating the corresponding organotriboronates, and consequently, cyclic boronic esters have been prepared, as they are generally more stable towards hydrolysis. The transesterification of tris(dimethoxyboryl)methane with ethylenglycol or pinacol provides a direct access to the cyclic esters ethylenglycolboryl and pinacolboryl [3], with the expected degree of stability (Scheme 2) [4].

**Scheme 3:**
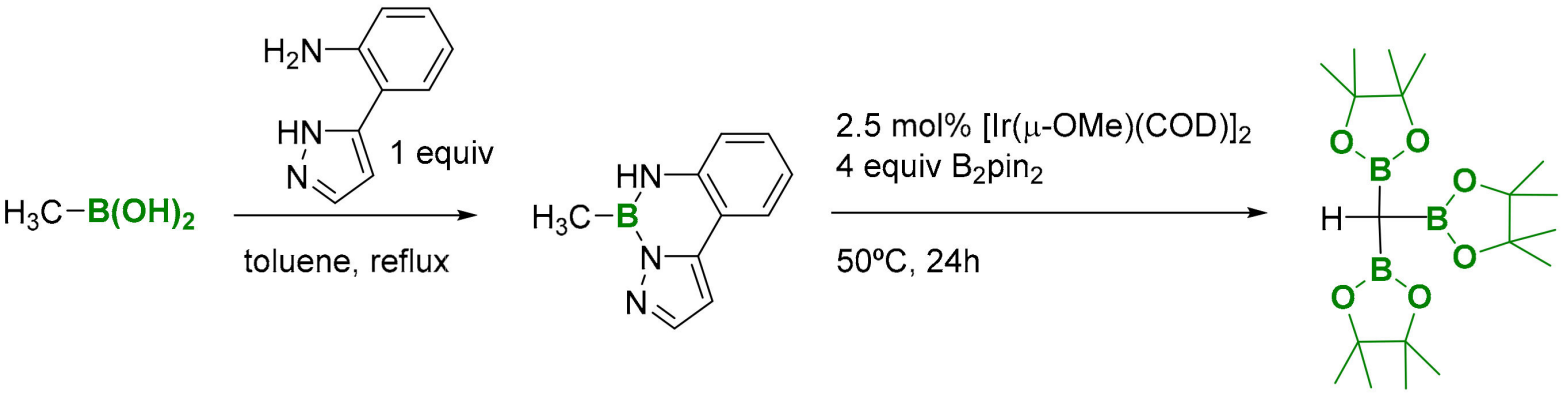
Synthesis of H(Bpin)_3_ from Me-B(OH)_2._

In particular, the synthesis of tris(pinacolboryl)methane has been recently redesigned by Suginome and co-workers, via the α,α-diborylation of pyrazolylaniline-modified methylboronic acid with B_2_pin_2_ in the presence of [Ir(μ-OMe)(COD)]_2_ as catalyst (Scheme 3) [5]. 

**Scheme 4:**
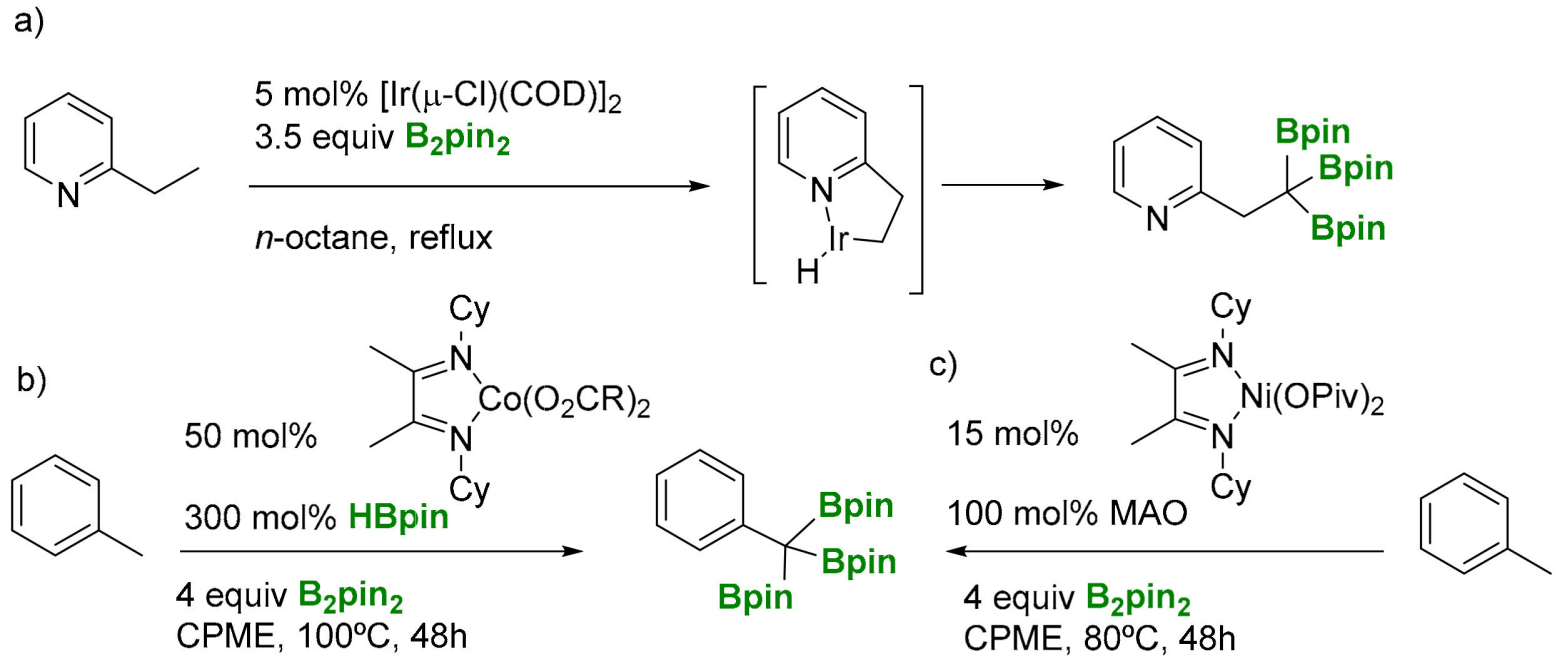
Transition metal catalytized C(sp^3^)–H triborylation at a single carbon.

Triborylalkane derivatives have been prepared via the triple borylation of terminal C(sp^3^)-H bonds with transition-metal complexes. Sato, Mita and co-workers determined the first catalytic C(sp^3^)–H triborylation at a single carbon with the assistance of a nitrogen directing group [6]. Model substrate 2-ethylpyridine forms a five-membered metallacycle intermediate, via C(sp^3^)–H activation with Ir from [Ir(μ-Cl)(COD)]_2_ catalyst, that subsequently reacts with 4 equiv of B_2_pin_2_ towards site-selective C(sp^3^)–H borylation leading to the desired triborylated product (Scheme 4a). Triborylation of terminal C(sp^3^)-H bonds in toluene was also observed by Chirik and co-workers by using 50 mol% of α-diimine cobalt bis(carboxylate) precatalysts and 4 equiv of B_2_pin_2_ (Scheme 4b) [7]. Similarly, α-diimine nickel bis(carboxylate) complex enables the selective preparation of benzyltriboronates via the triborylation of benzylic C−H bonds (Scheme 4c) [8].

**Scheme 5:**
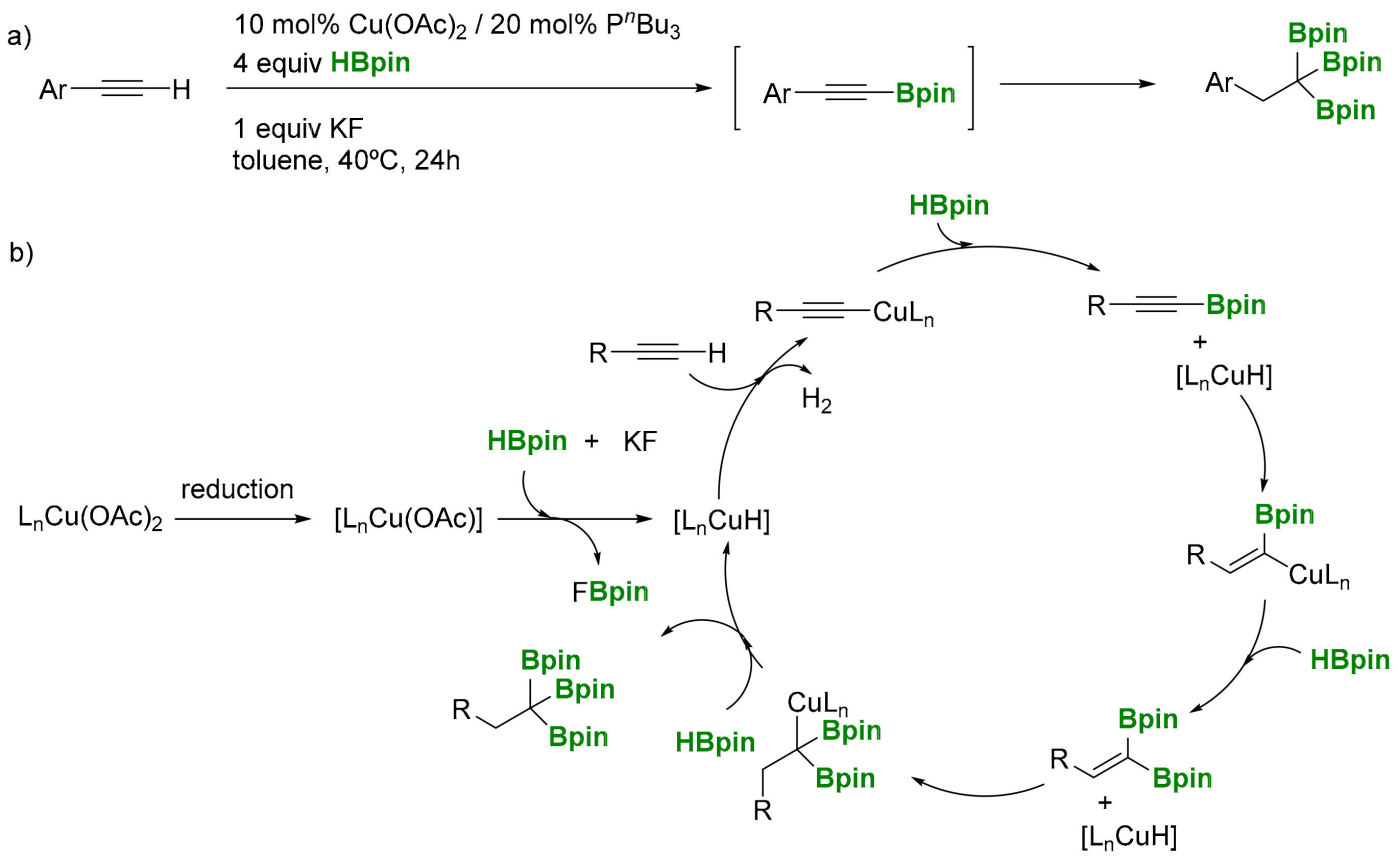
(**a**) sequential dehydrogenative borylation/hydroboration of terminal alkynes; (**b**) mechanistic proposal.

**Scheme 6:**
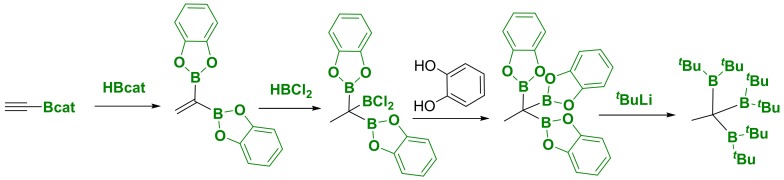
Synthesis of 1,1,1-tris(catecholboryl)alkane and 1,1,1-tris[di(tert-butyl)boryl]ethane.

The sequential dehydrogenative borylation/hydroboration of terminal alkynes with Cu complexes contributes to the expansion of the triborylalkane derivatives synthesis. Marder and co-workers have developed a one-step synthesis via the dehydrogenative borylation of alkynes followed by the double hydroboration of 1-alkynylboronate intermediate with HBpin (HBpin = pinacolborane) catalyzed by Cu(OAc)_2_ (Scheme 5a) [9]. Interestingly, other copper sources, such as CuCl_2_, CuCl, and Cu(OTf)_2_, resulted in being inactive in this transformation. The base KF was essential in a stoichiometric manner, presumably by promoting the Cu-H formation in an early step of the catalytic cycle (Scheme 5b). This strategy complements a pioneer attempt by Marder and co-workers to perform the Rh-catalyzed reaction of ethynylarenes with HBcat (HBcat = catecholborane), to obtain the corresponding tris(catecholboryl)alkane, via vinylboronate intermediate [10]. Catechol-substituted monoborylacetylene could be regioselectively hydroborted via the transition-metal free syn-addition of catecholborane favoring the formation of the 1,1-diborylalkene, that can be further hydroborated selectively with HBCl_2_ to provide the corresponding 1,1,1-triborylethane. The subsequent substitution of Cl atoms by cathecol leads to the desired tris(catecholboryl)ethane [11]. Further treatment of tris(catecholboryl)ethane with ^t^BuLi results in the formation of 1,1,1-tris[di(tert-butyl)boryl]ethane (Scheme 6).

**Scheme 7:**
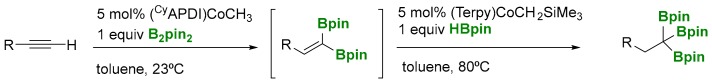
Cobalt-catalyzed dehydrogenative 1,1-diboration of terminal alkynes.

Alternatively, Chirik and co-workers have demonstrated that a cyclohexylsubstituted pyridine(diimine) cobalt methyl complex allows the dehydrogenative 1,1-diboration of terminal alkynes with B_2_pin_2_ at room temperature. This 1,1-diborylalkene intermediate can accomplish a consecutive cobalt-catalyzed hydroboration with HBpin to generate the desired of 1,1,1-triborylalkane (Scheme 7) [12]. The methodology is described as a one pot-sequential 1,1-diboration of terminal alkynes with B_2_pin_2_, followed by hydroboration with HBpin, although two different types of cobalt complexes are used for each step.

**Scheme 8:**
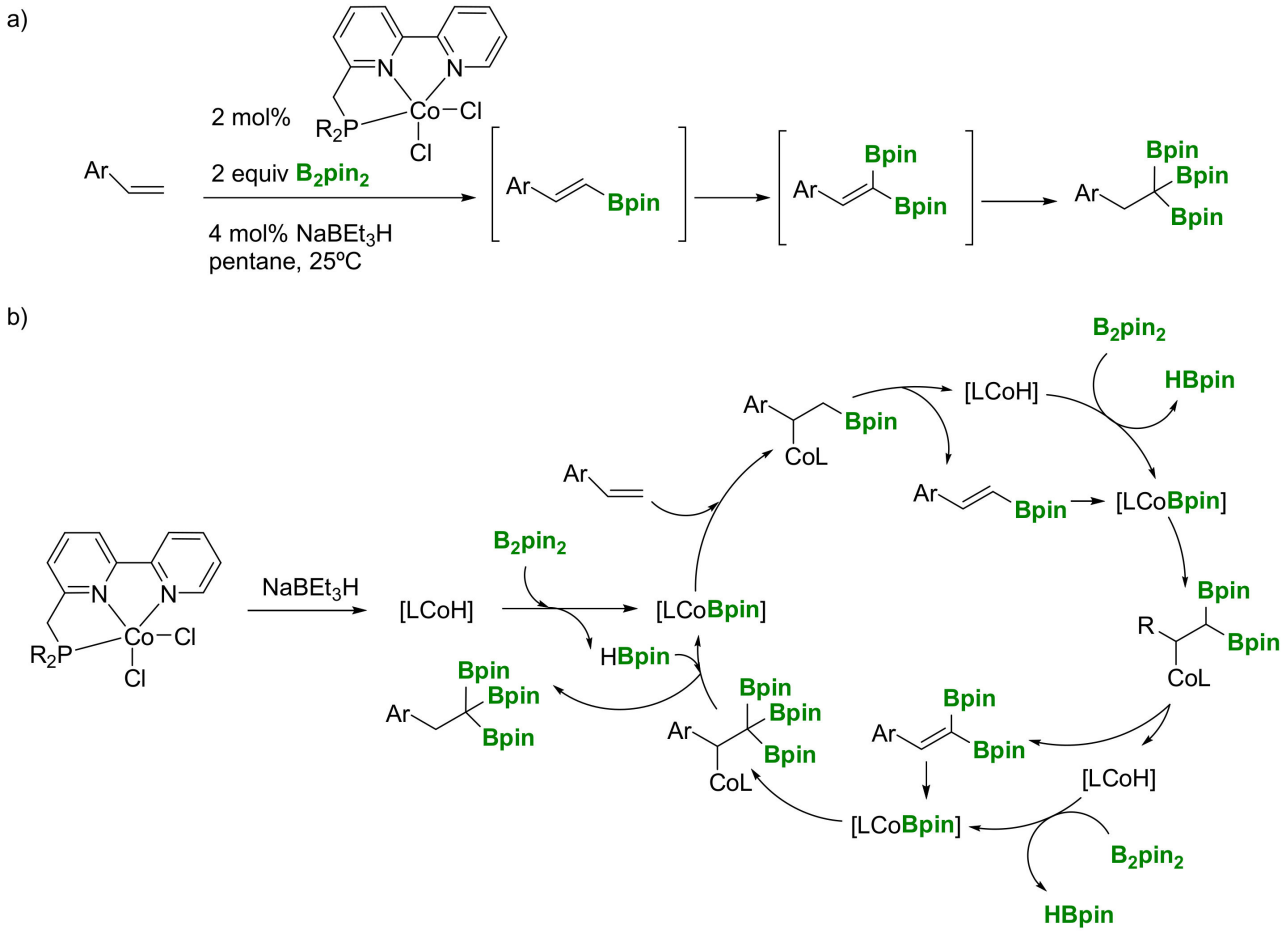
(**a**) Co(I)-catalyzed double dehydrogenative borylation of vinylarenes; (**b**) mechanistic proposal.

Huang and co-workers have developed a Co(I)-catalyzed double dehydrogenative borylation of vinylarenes with B_2_pin_2_ to generate a 1,1-diborylalkene intermediate, which undergoes hydroboration with pinacolborane to give 1,1,1-tris(boronates) in high yield (Scheme 8a) [13]. The dehydrogenative borylation of alkenes leading to vinylic boron compounds was first reported in 1992 [14], and it is understood as being the result of a catalytic hydroboration of an alkene with a monohydrideborane, followed by the elimination of hydrogen. However, Huang and co-workers have postulated a mechanism where the dehydrogenative borylation occurs by the insertion of vinylarenes into the Co−B bond to generate a β-boryl-substituted Co(I) species, which undergoes β-hydride elimination to give monoborylalkenes and the Cu-H intermediate that regenerates the Co-B catalytic species via σ-bond methatesis with B_2_pin_2_, maintaining the same type of reactivity along the catalytic cycle (Scheme 8b). As a limitation, this protocol cannot be applied for 1,2- and 1,1-disubstituted vinylarenes as well as vinylalkanes, since 1,1,1-tris(boronates) were not formed.

### 2.2. Synthetic Applications

**Scheme 9:**
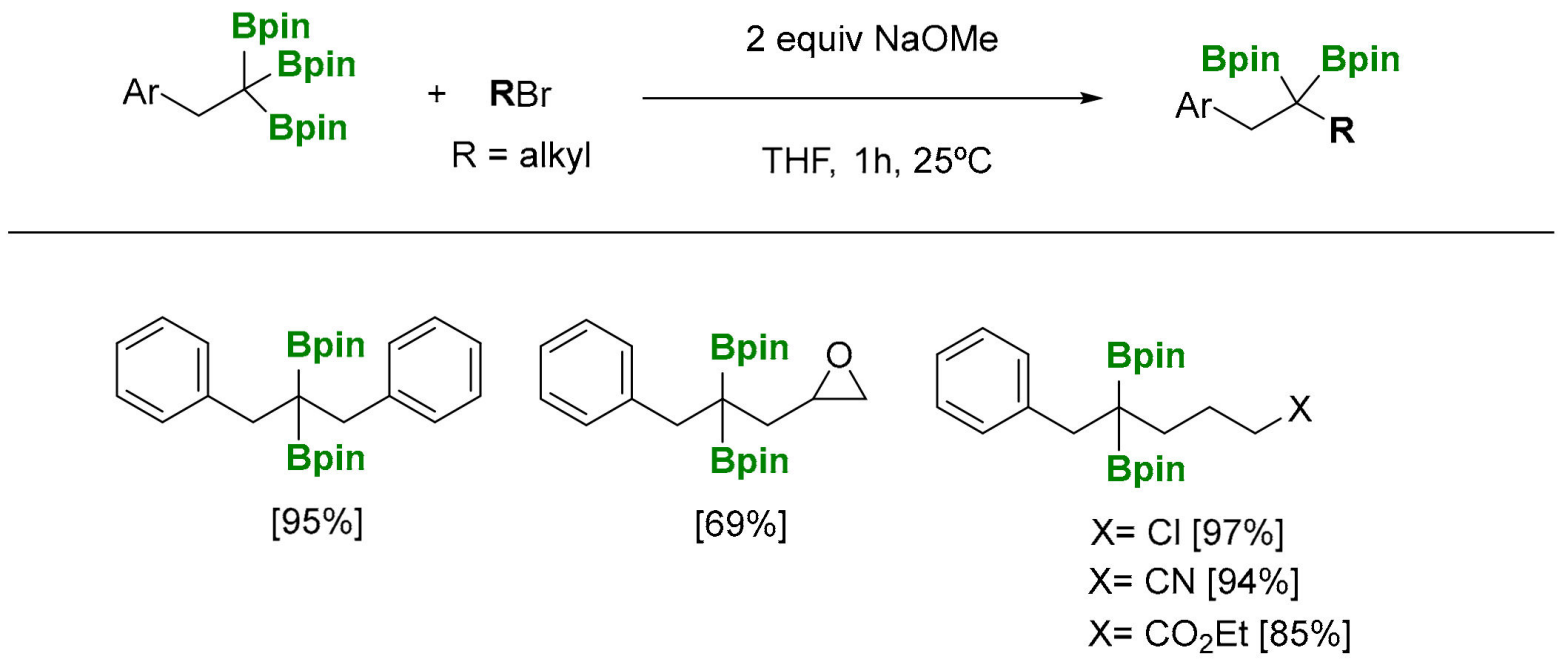
Reactivity of 1,1,1-tris(pinacolboryl)alkanes with RBr.

The reactivity of 1,1,1-tris(pinacolboryl)alkanes has been launched by alkoxide-assisted deborylative pathways, via α-bis(boryl) carbanion formation, that can be eventually trapped by different electrophiles [4]. The deborylative alkylation of 1,1,1-tris(boronate) products has been applied to the synthesis of internal geminal bis(boronates), by reaction with alkyl bromides in the presence of NaOMe (1.5 equiv), at 25 °C (Scheme 9) [13]. Interestingly, 1,1,1-tris(boronates) seems to be more reactive for deborylative alkylations than the analogue 1,1-bis(boronates), as the reactions with the latter require the use of an excess of NaO^t^Bu (3 equiv) [15]. The reaction is compatible with diverse functionalities in the electrophiles, including cyanide, propylene epoxide, ester, halides and amines.

**Scheme 10:**
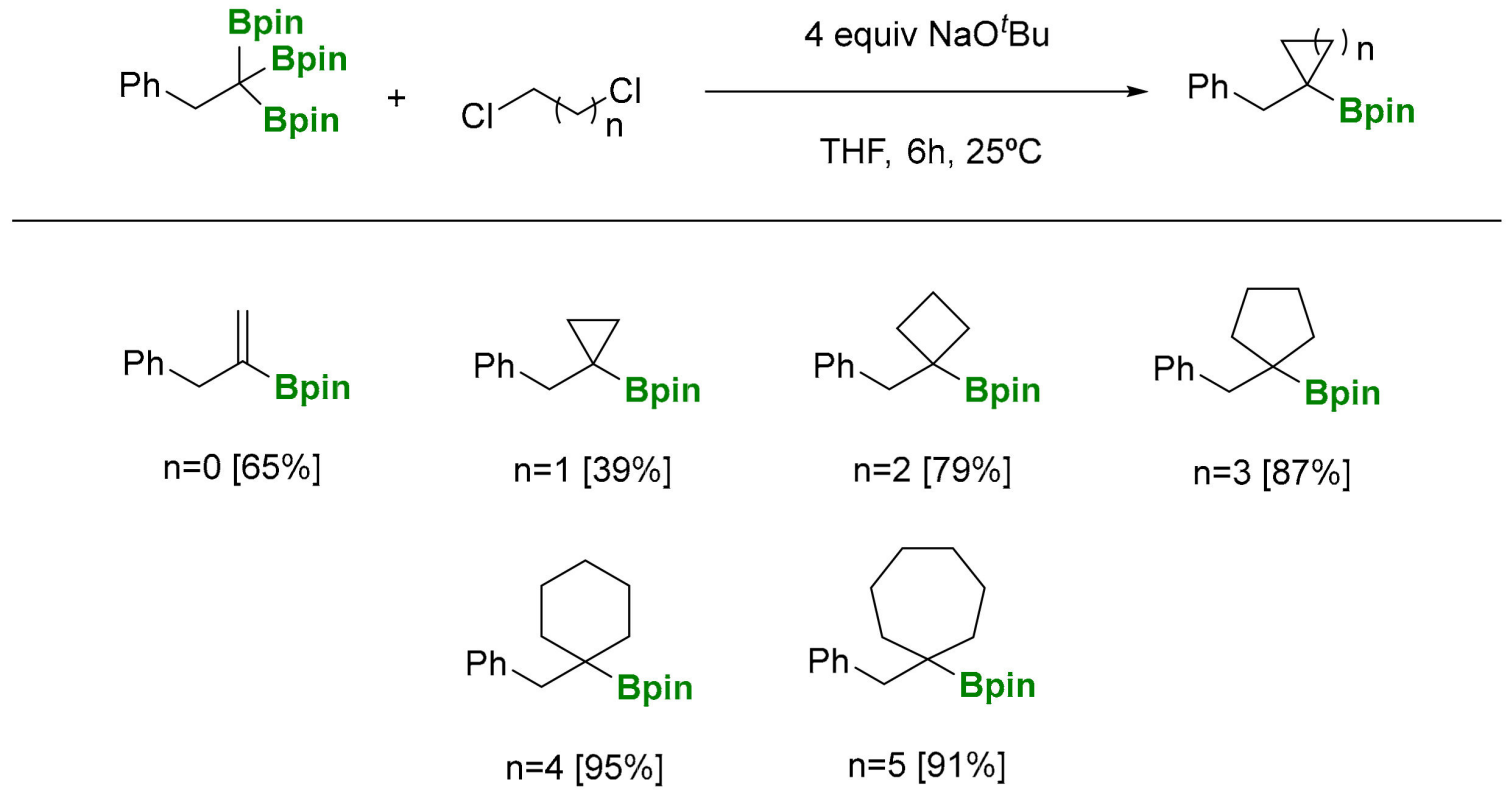
Double deborylative alkylation of 1,1,1-tris(pinacolboryl)alkanes.

**Scheme 11:**
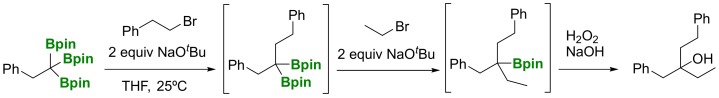
Sequential deborylative alkylations, followed by in situ oxidation with H_2_O_2_/NaOH, towards tertiary alcohols.

Double deborylative alkylation delivers tertiary boronic esters, as well as carbocyclic derivatives. Using 1,n-dihalides as electrophiles and NaO^t^Bu (4 equiv), the base-assisted deborylation acts twice on the triborylated reagent, to facilitate the formation of α-vinylboronates (when n = 0) and cyclic organoboronates (when n = 1–5) at room temperature within 6h (Scheme 10) [9]. Following the same synthetic strategy, different alkyl groups can be introduced in a stepwise manner through sequential deborylative alkylations, providing access to tertiary boronic esters, which, after in situ oxidation with H_2_O_2_/NaOH, furnished tertiary alcohols containing three different alkyl groups in moderate yield (Scheme 11) [9]. 

**Scheme 12:**
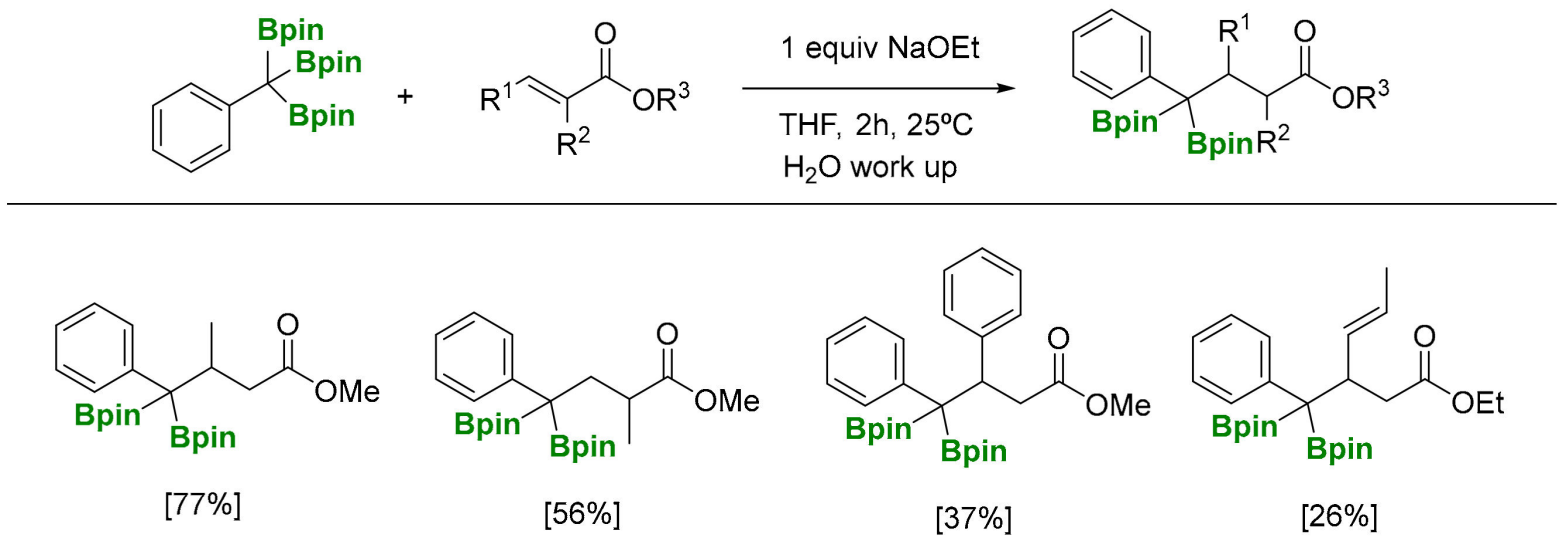
Deborylative conjugate addition of 1,1,1-tris(pinacolboryl)alkanes.

**Scheme 13:**
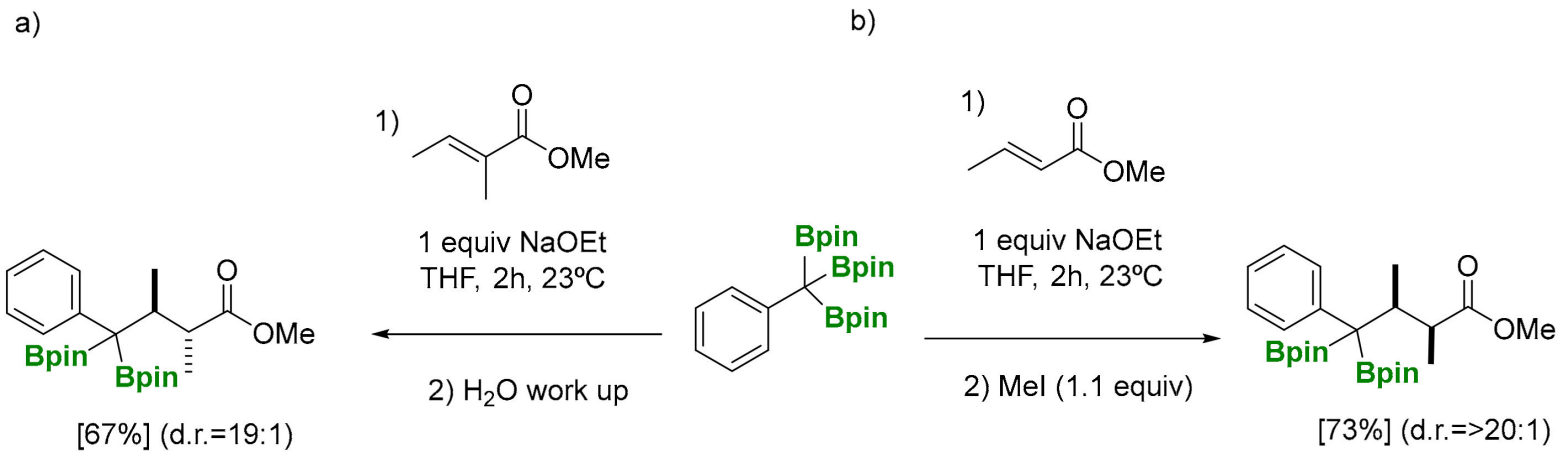
(**a**) diastereoselective deborylative conjugate addition of 1,1,1-tris(pinacolboryl)alkanes; (**b**) diastereoselective sequential deborylative conjugate addition / electrophilic methylation.

The deborylative conjugate addition of 1,1,1-tris(pinacolboryl)alkanes has been pursued through a NaOEt (1 equiv) assisted reaction to generate benzylic α-diboryl anions that can act as soft nucleophiles to be added to (E)-methylcrotonate, at room temperature, followed by aqueous work up (Scheme 12) [8]. Products formed from the deborylative conjugate addition to phenyl-substituted (E)-methyl cinnamate, as well as α,β,γ,δ-unsaturated ethyl sorbate, suffered from a competitive 1,2-addition to the more electron deficient enoates (Scheme 12) [8]. The reaction can be diastereoselective for α-subtituted substrates, such as methyl (E)-2-methylbut-2-enoate, in favor of the anti diastereomer. The stereocontrol at the α-position of the ester group has been suggested to occur during the protonation when the reaction is quenched (Scheme 13a) [8]. Remarkably, when the deborylative conjugate addition of the benzylic α-diboryl anion to (E)-methylcrotonate was quenched with iodomethane, the resulting product was isolated in >20:1 as the syn diastereomer (Scheme 13b) [8], highlighting the exclusive ability of boronate groups to act as masking groups for soft nucleophilic anions and also as elements for substrate-controlled diastereoselectivity.

**Scheme 14:**
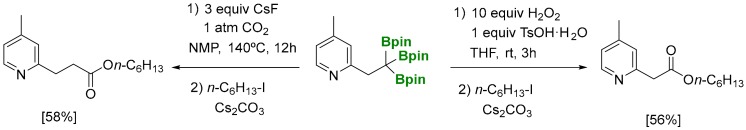
Carboxylation of 1,1,1-tris(pinacolboryl)alkanes.

The carboxylation of 1,1,1-tris(pinacolboryl)alkanes, using CsF as a mild activator for deborylation [16], has been explored under 1 atm of CO_2_, providing the corresponding carboxylated products in moderate yields, after esterification with RI (Scheme 14) [6]. In addition, the sequence based on the trioxidation of 1,1,1-tris(pinacolboryl)alkanes using H_2_O_2_ in the presence of an acidic additive such as TsOH·H_2_O prevents protodeborylation pathways, and n-C_6_H_13_-I allows the isolation of hexyl 2-(4-methylpyridin-2-yl)acetate in moderate yield. Both methods’ results are useful, since carboxylic acids with complementary chain lengths can be prepared by selecting the appropriate reagents to interact with the tris(boryl)alkane.

**Scheme 15:**
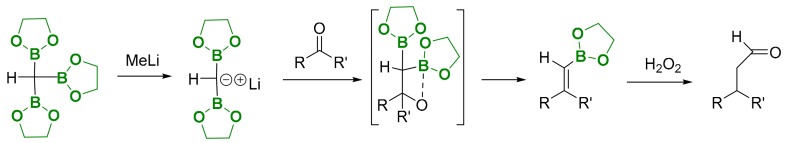
Deborylative Boron–Wittig olefination from tris(boryl)alkanes.

Other interesting reactivity in C-C bond formation from tris(boryl)alkane involves deborylative Boron–Wittig olefination, via the B-O elimination pathway. Originally, the proof of concept was established by the addition of methyllithium to tris(ethylenedioxyboryl)methane, to generate in situ the corresponding bis(ethylenedioxyboryl)methide salt, that interacts with aldehydes or ketones to give the corresponding alkene monoboronic esters (Scheme 15) [17,18].

**Scheme 16:**
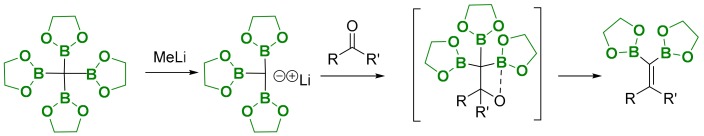
Deborylation of tetra(boryl)methane and condensation with aldehydes and ketones.

The analogue 1,1,1-tris(boryl)methide salt has exclusively been prepared via base-assisted deborylation of the corresponding tetra(boryl)methane (C(BO_2_C_2_Me_4_)_4_) [19], promoting the condensation with aldehydes and ketones to obtain 1,1-gem-diborylalkenes (Scheme 16) [20].

The reactivity developed from 1,1,1-tris(boryl)alkanes offers a significant number of applications, since their activation in the presence of bases generates the corresponding carbanions, which are strongly stabilized by delocalization, with vacant p-orbitals of the remaining two boron atoms. The possibility to perform further functionalization of the remaining C-B bonds eventually leads to new strategic synthetic protocols, emphasizing the straightforward methodology, as well as the smooth reaction conditions.

## 3. 1,2,3-Tri(boryl)alkanes

### 3.1. Synthetic Approaches

**Scheme 17:**
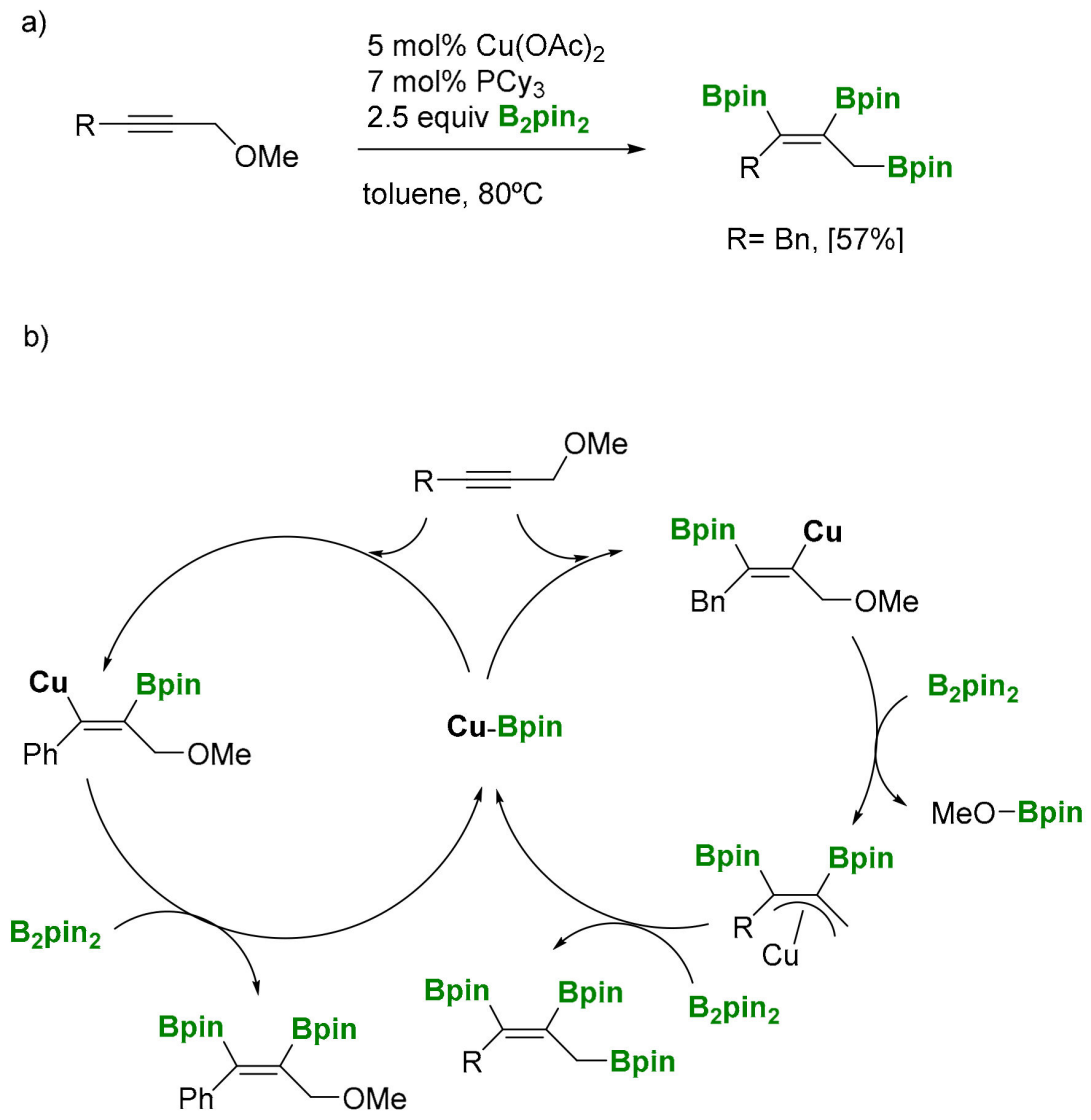
(**a**) Borylcupration of alkyl-substituted propargyl ethers; (**b**) mechanistic proposal.

The introduction of three vicinal boryl moieties in a one-pot protocol represents a challenging reaction. The triboration of alkynes towards 1,2,3-triborated compounds has been accomplished by Yoshida and co-workers [21] via the Cu–PCy_3_ catalysed borylcupration of alkyl-substituted propargyl ethers (Scheme 17a). This approach demonstrates the easy access to multifunctinalized organoboron compounds that contain two alkenyl boryl and one allylboryl moiety. Mechanistically, it has been suggested that alkyl-substituted propargyl ethers insert regioselectively into Cu-B bonds generating the alkenyl copper where Cu is located next to OMe group. Subsequently, σ-bond methatesis with B_2_pin_2_ facilitates the formation of π-allyl species that after a second σ-bond methatesis with the diboron reagent provides the triborated product (Scheme 17b). However, phenyl-substituted propargyl ethers react towards the diborated product, presumably as a consequence of the regioselective alkenyl copper species formation, with Cu next to Ph group, and eventual σ-bond metathesis with B_2_pin_2_ (Scheme 17b).

**Scheme 18:**
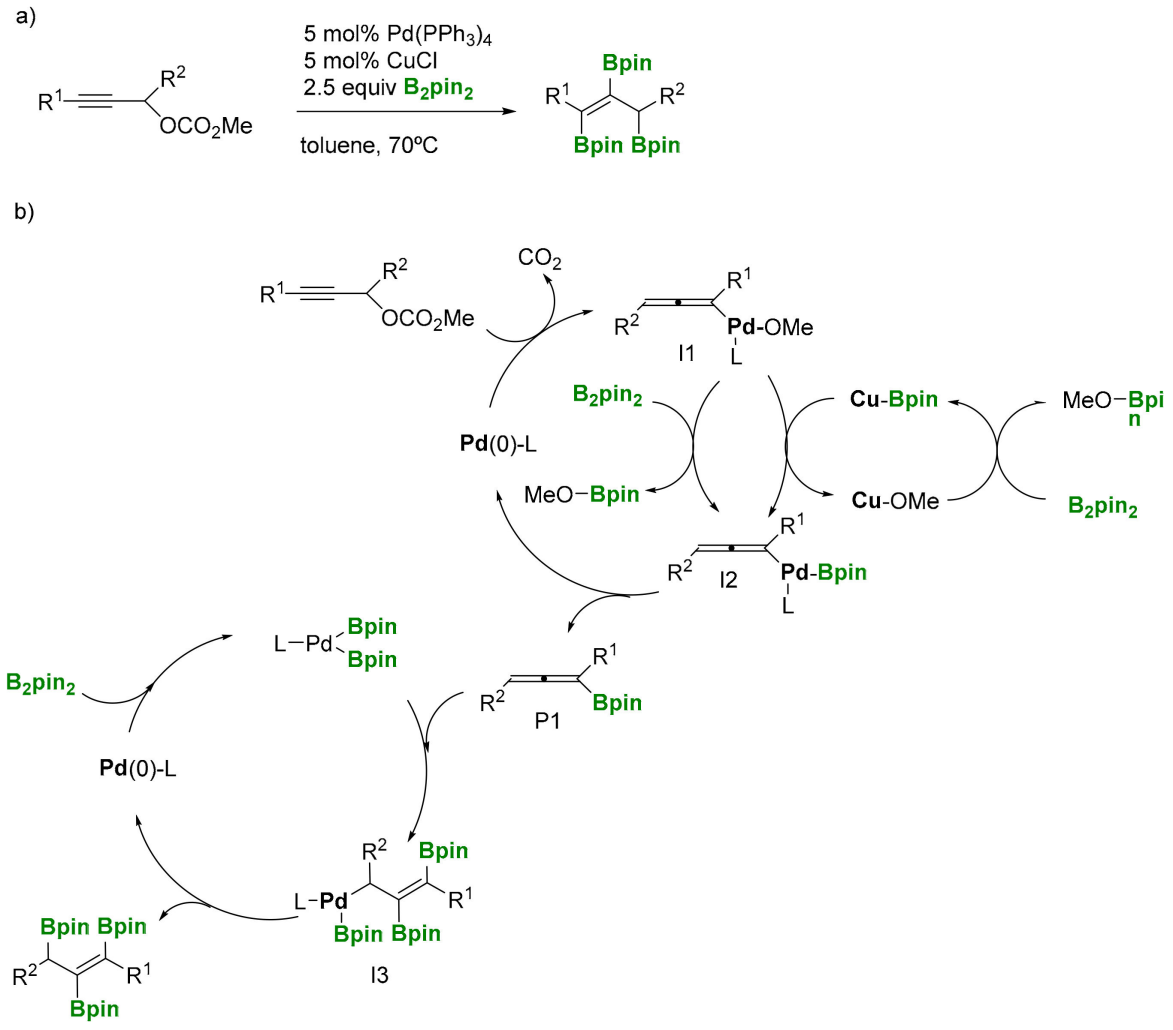
(**a**) 1,2,3-Triboration of propargylic carbonates; (**b**) mechanistic proposal.

Alternatively, Ma and co-workers [22] developed a highly selective 1,2,3-triboration of propargylic carbonates towards (E)-propen-1,2,3-triboronates, by the catalytic systems Pd(PPh_3_)_4_ and CuCl as co-catalyst (Scheme 18a). Mechanistically, two catalytic cycles have been suggested. The first one is based on the Pd(0) catalyzed S_N_2′-type oxidative addition of propargylic carbonates to give 1,2-allenyl palladium intermediate I1 (Scheme 18b), with the concomitant release of CO_2_. In the following step, I1 undergoes transmetallation with Cu-Bpin species, previously formed from Cu-OMe and B_2_pin_2_, in order to generate the intermediate I2. Subsequent reductive elimination allows the formation of 1,2-allenyl boronate P1, that releases from the catalytic cycle 1. Interestingly, it has been suggested that P1 might insert in a Pd-B bond in catalytic cycle 2, producing η^1^-allylic intermediate I3, which undergoes reductive elimination to afford the final triborated product with exclusive (E)- stereoselectivity (Scheme 18b).

**Scheme 19:**
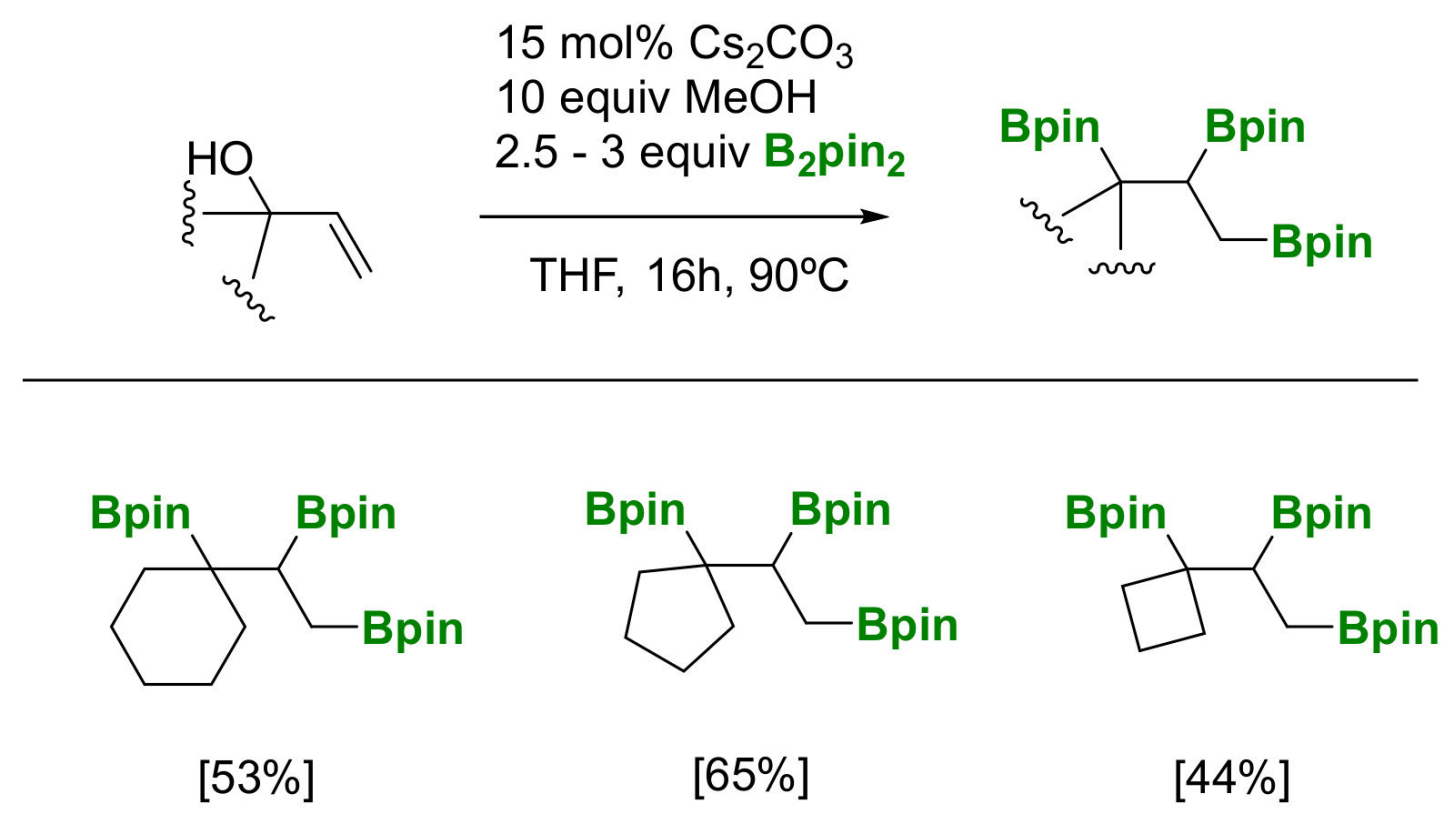
Transition metal-free one-pot allylic borylation/diboration sequence.

**Scheme 20:**
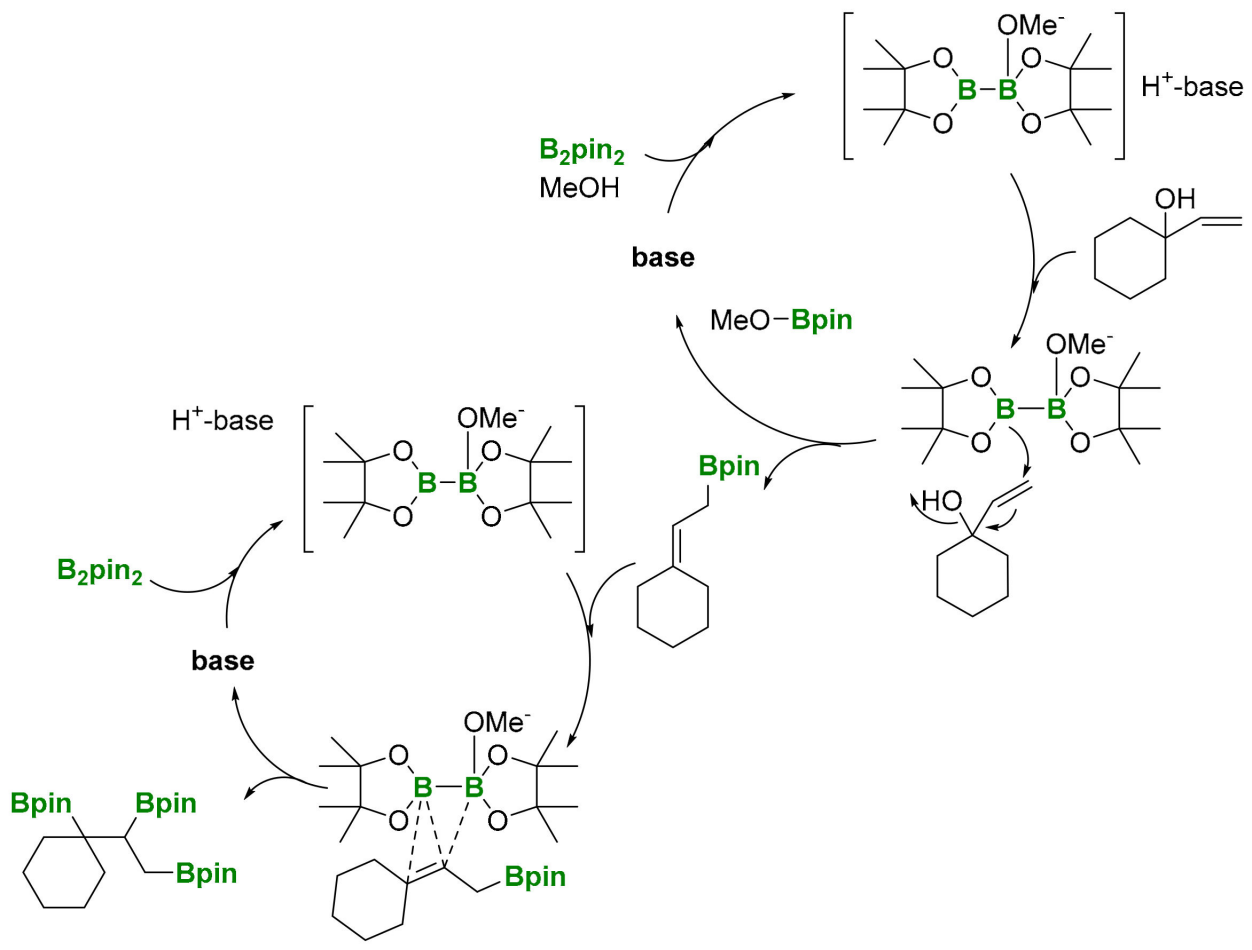
Mechanistic proposal of transition metal-free 1,2,3-triboration of allylic alcohols.

All the previous efforts devoted to introducing three vicinal C–B bonds into a one-pot sequential reaction required transition metal complexes to activate the borane reagent and facilitate the addition to the unsaturated substrates. However, Fernández, Szabó and co-workers [23] were first to observe the possibility of conducting the 1,2,3-triboration reaction in a transition-metal free context, using allylic alcohols as substrates and conducting a one-pot allylic borylation/diboration sequence (Scheme 19). The reaction takes place in the presence of catalytic amount of base Cs_2_CO_3_, that contributes to the generation of MeO^-^ from MeOH. The methoxy group activates the B_2_pin_2,_ through intermediate [Hbase][MeOB_2_pin_2_], and promotes the nucleophilic attack of the Bpin moiety to the terminal position of the tertiary allylic alcohol (Scheme 20).

The initial mechanism of this reaction can be described as an S_N_2′-type process. In the absence of MeOH or base, the reaction does not take place. After the transition-metal free allylic borylation, the allylic boronate product can interact with the [Hbase][MeOB_2_pin_2_] adduct and complete the diboration reaction [24], to generate the 1,2,3-triborated product (Scheme 20). This unprecedented tandem allylic borylation/diboration reaction in the absence of transition-metal catalysts had not been observed before and served as the basis of a new methodology towards polyborated compounds.

**Scheme 21:**
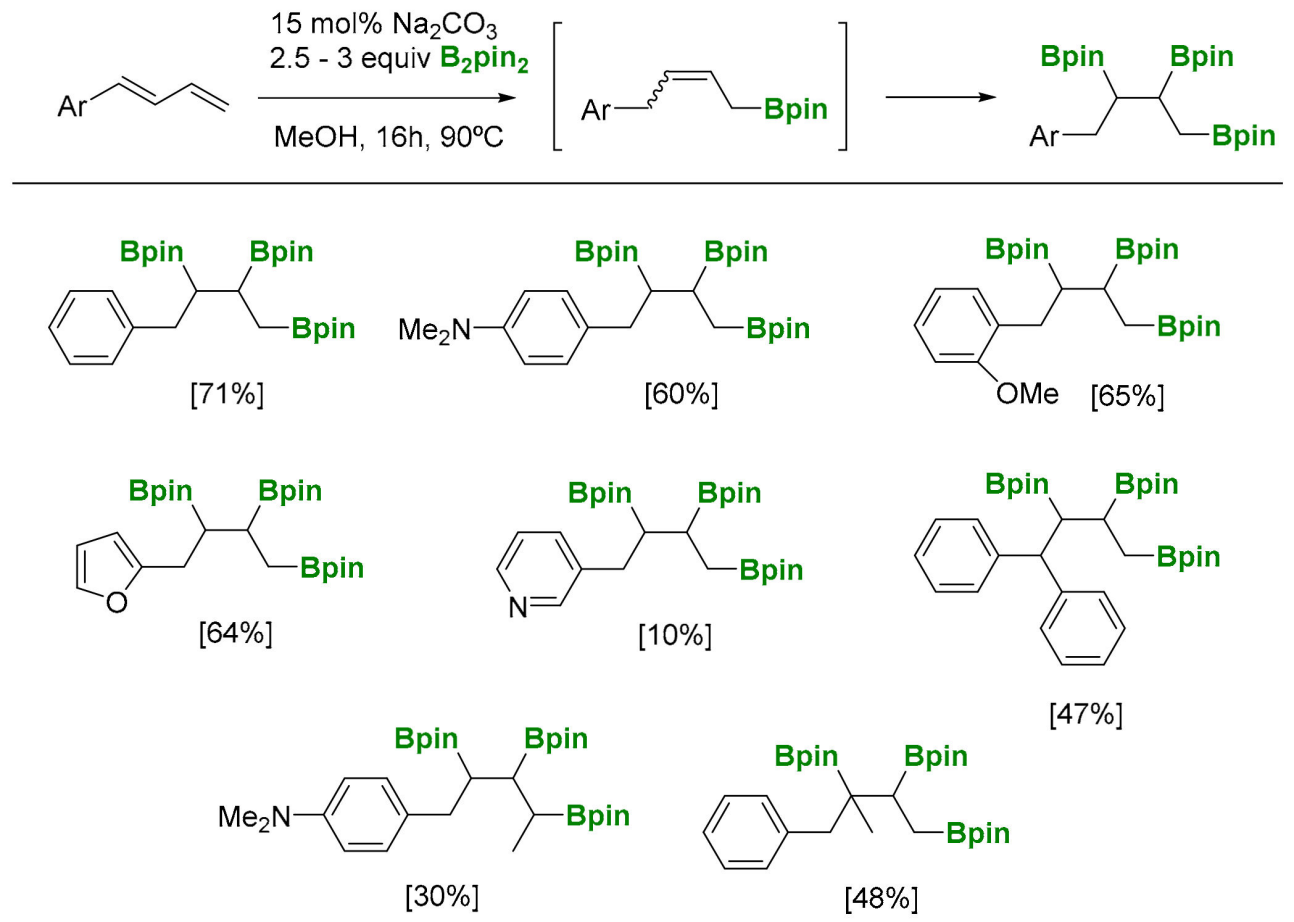
Transition metal-free 1,2,3-triboration of conjugated dienes.

Fernández and co-workers also explored the viability of performing a direct 1,2,3-triboration reaction in a transition-metal free scenario via the 1,4-hydroboration of 1,3-dienes [25], followed by the in situ diboration reaction of the resultant allylic boronate (Scheme 21) [26]. This work represents an unprecedented tandem 1,4-hydroboration/diboration reaction, that is generalized for terminal and internal 1,3-dienes.

**Scheme 22:**
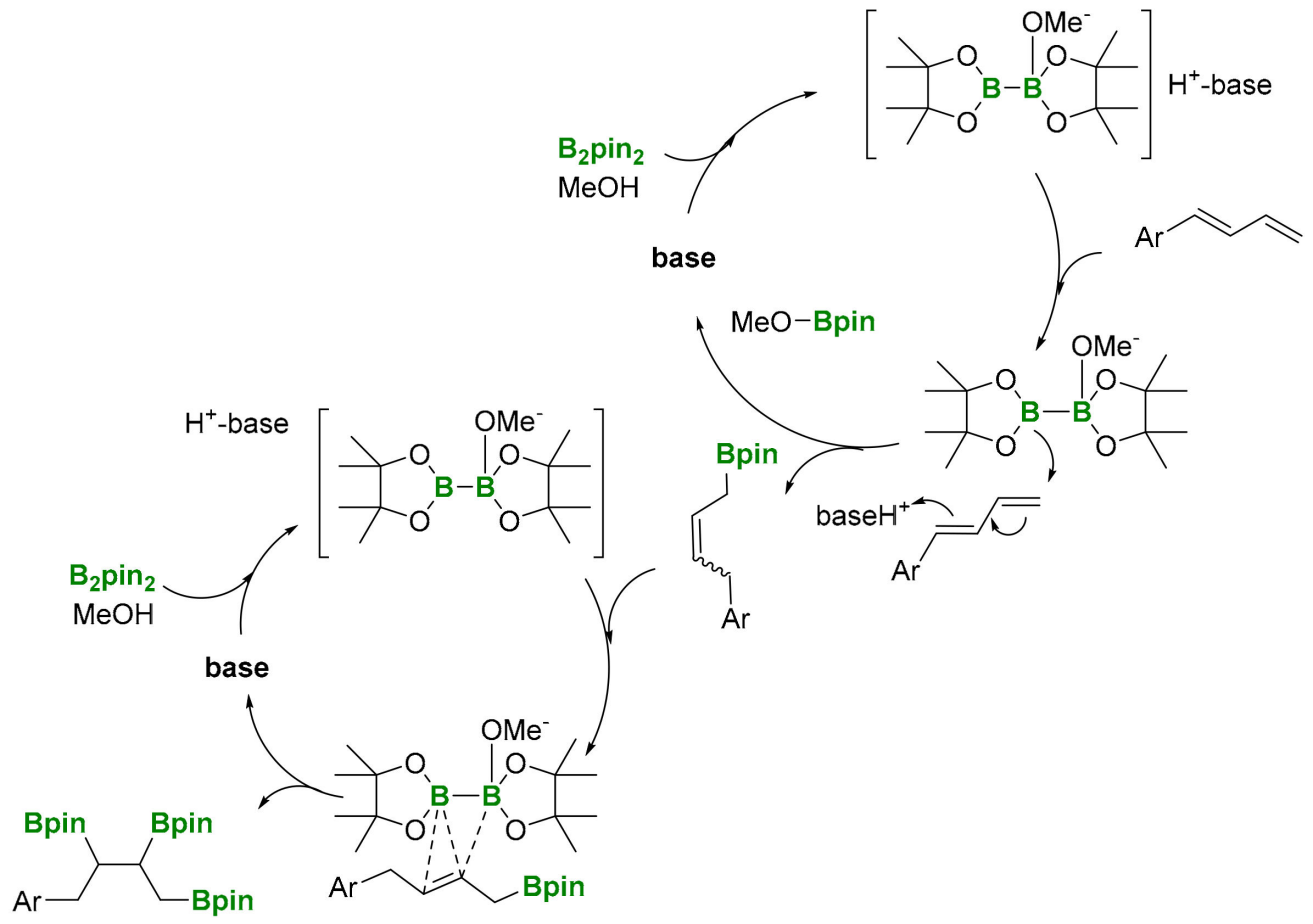
Mechanistic proposal of transition metal-free 1,2,3-triboration of conjugated dienes.

The nucleophilic attack of the Bpin moiety from [Hbase][MeOB_2_pin_2_] takes place to the less hindered position of the conjugated dienes throughout a S_N_2′-borylative process. The resulting allylic boronate product conducts a transition-metal free diboration reaction of the internal C=C bond, giving access to the 1,2,3-triborated product (Scheme 22) [26].

### 3.2. Synthetic Applications

**Scheme 23:**
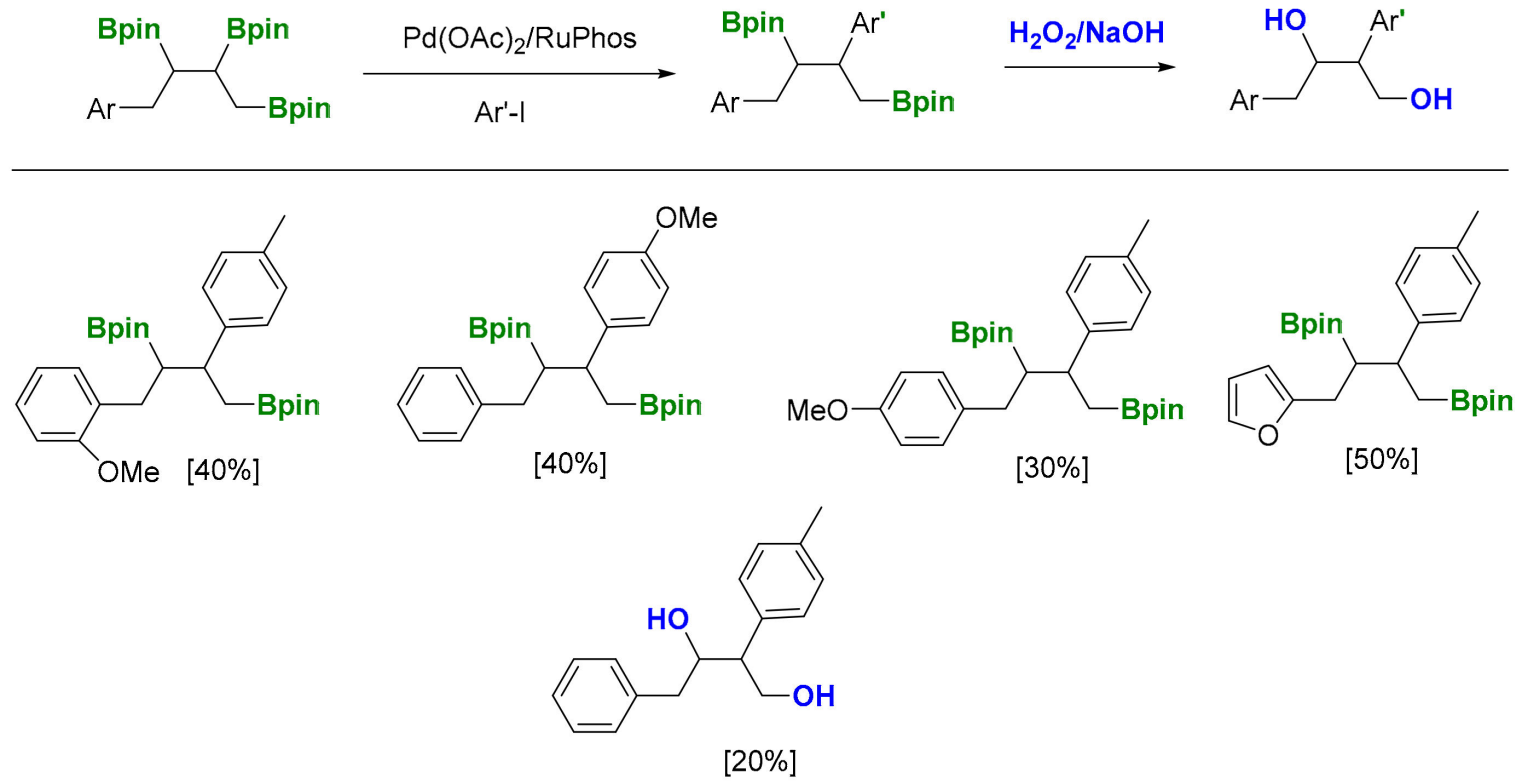
Selective polyfunctionalization of 1,2,3-triborated products.

The polyfunctionalization of 1,2,3-triborated products can be accomplished throughout the cross-coupling reaction. Interestingly, in the presence of the 1 equiv of aryliodide and Pd complex, it was possible to exclusively functionalize the internal C-B bond of the triborated product (Scheme 23) [26]. The reaction was general for para- and ortho-substituted aryl systems, as well as for furyl groups. The in situ oxidation of the resulting 1,3-diborated products, in the presence of H_2_O_2_/NaOH, allowed access to 1,3-butanediol 2,3-diaryl systems in a straightforward manner (Scheme 23) [26].

**Scheme 24:**
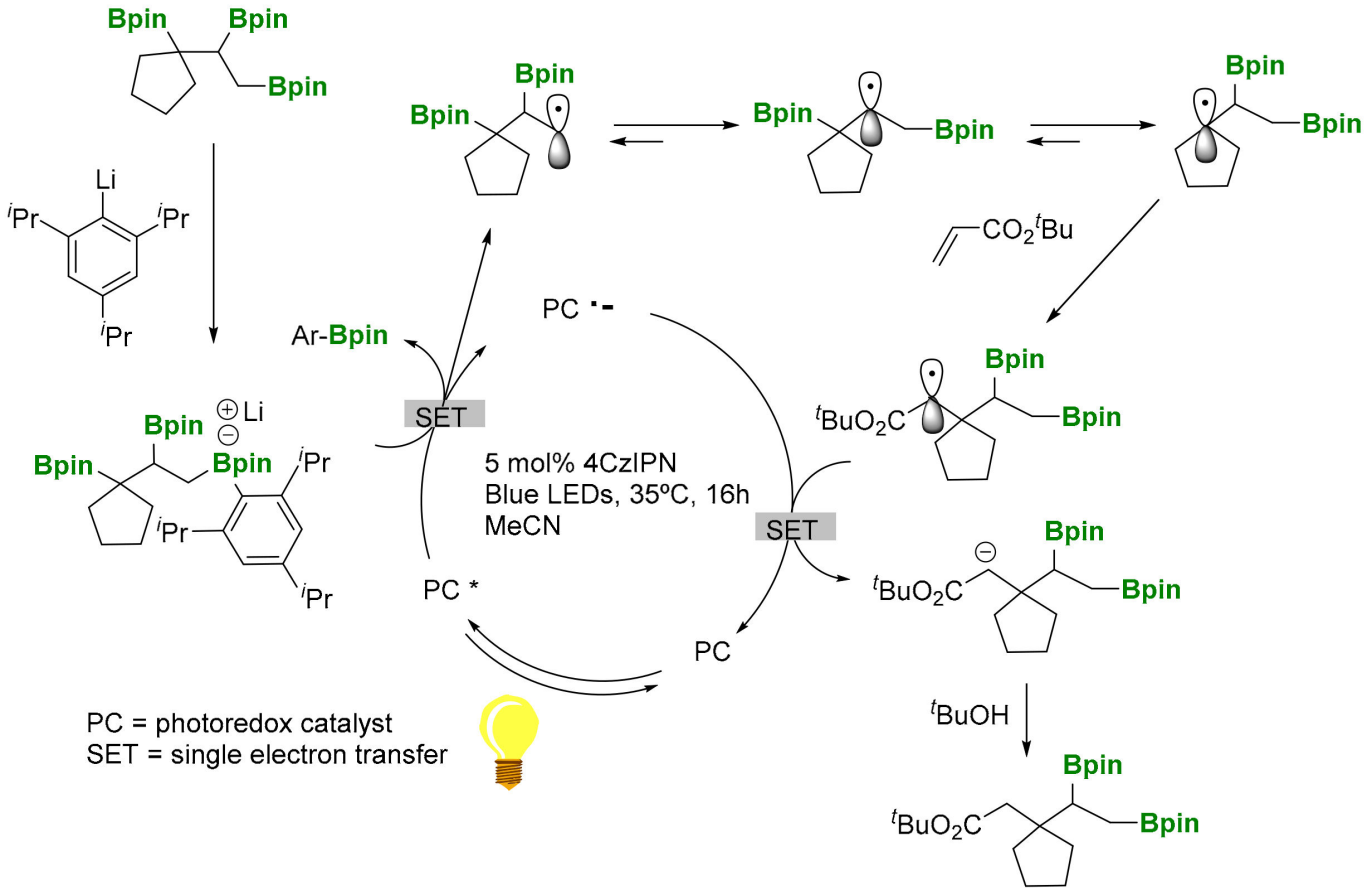
Photoredoxcatalyzed mono-deboronation of 1,2,3-triborated products.

Aggarwal and co-workers have reported an alternative functionalization of 1,2,3-triborated compounds following a photoredoxcatalyzed mono-deboronation. Initially the reaction proceeds through the generation of primary β-boryl radicals that undergo a rapid 1,2-boron shift to form thermodinamically favored secondary followed by a subsequent 1,2-boron shift towards tertiary radicals (Scheme 24). This protocol allows for the selective transformation of the more hindered boronic ester from the 1,2,3-triborated substrate [23], conducting its radical addition to tert-butyl-acrylate [27]. The product is obtained with high selectivity, highlighting the thermodynamic control that favors the most stabilized radical center as the site of the reaction.

## 4. 1,1,2-Tri(boryl)alkanes

### 4.1. Synthetic Approaches

**Scheme 25:**
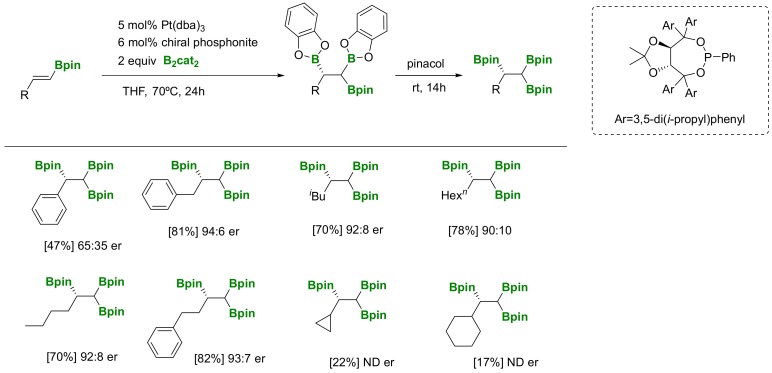
Synthesis of 1,1,2-tri(boryl)alkanes via bis(catecholato)diboron.

The preparation of versatile 1,1,2-tri(boryl)alkanes has been conducted via the Pt-catalyzed diboration of vinylboronates, (Scheme 25) [28]. Morken and co-workers employed chiral phosphonite to induce enantioselectivity along the internal C-B bond formation in the presence of Pt(dba)_3_ and bis(catecholato)diboron. Further treatment of the triborated product with pinacol, allowed the purification of the desired stable products with moderate to high enantioselectivity. The reaction has been considered of general application, although limitations are related to α-substituted substrates that suffered from lower reactivity and selectivity [28].

**Scheme 26:**
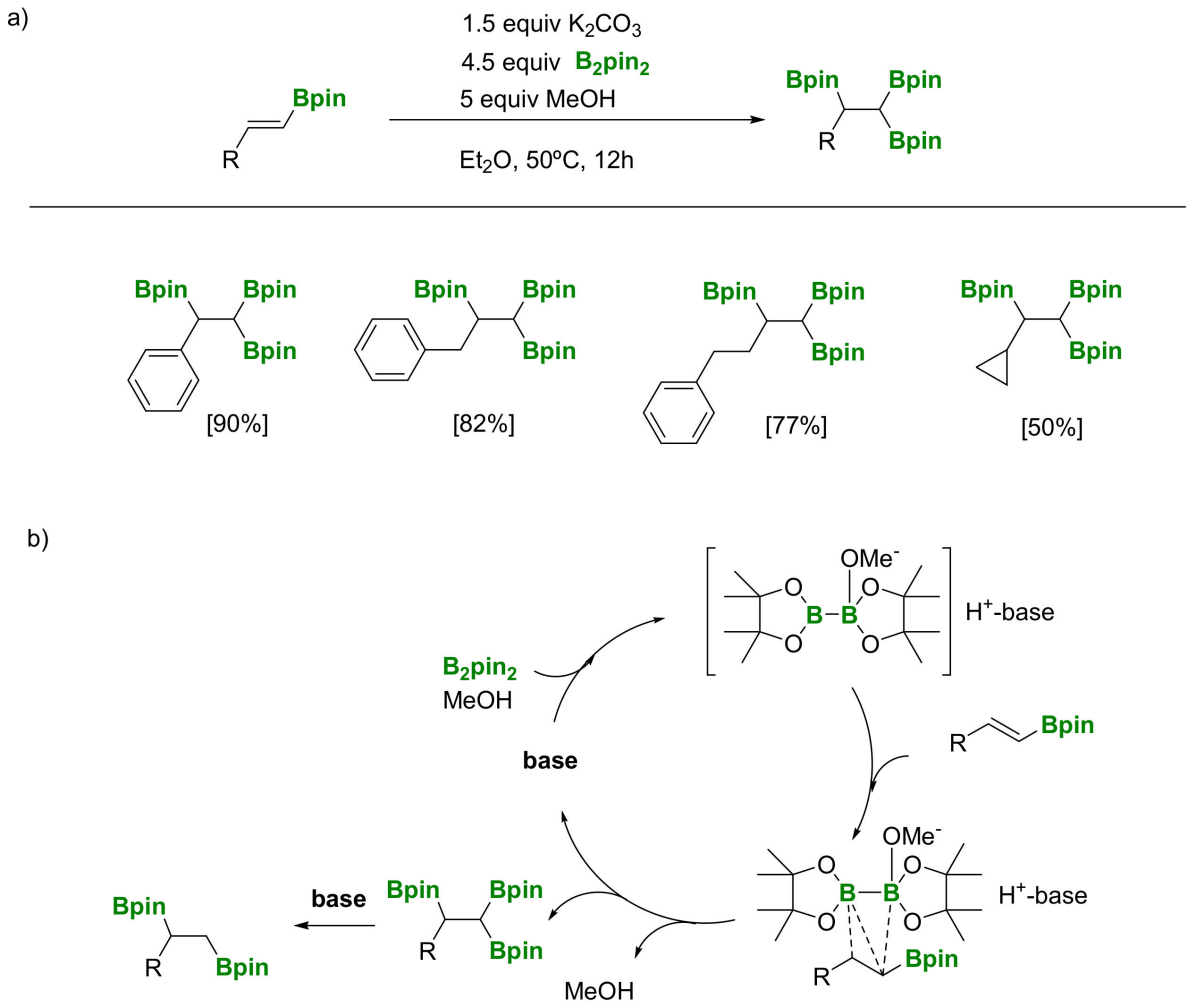
(**a**) transition-metal free 1,1,2-tris(boryl)alkane synthesis; (**b**) mechanistic proposal.

Another approach, launched by Song and co-workers [29], involves transition-metal free base-controlled 1,1,2-tris(boryl)alkane synthesis via diboration of alkenylboronates with B_2_pin_2_ (Scheme 26a). The reaction conditions for the synthesis of 1,1,2-tris(boryl)alkane were strictly optimized, since the corresponding 1,2-di(boryl)alkanes can also be formed as byproducts, depending on the base used. K_2_CO_3_ (1.5 equivalents) resulted in the optimized one, although 4.5 equivalents of B_2_pin_2_ were required. The substrate scope is broad and several functionalized terminal alkynes, such as propargyl amides and propargyl sulfonamides, are compatible. From a mechanistic point of view, a nucleophilic attack from the adduct [Hbase][MeOB_2_pin_2_], (generated in situ from B_2_pin_2/_base/MeOH) to the alkenylboronate substrate (Scheme 26b) has been suggested, as it was described on the transition-metal free diboration of alkenes developed by Fernández and co-workers in 2011 [24].

### 4.2. Synthetic Applications

**Scheme 27:**
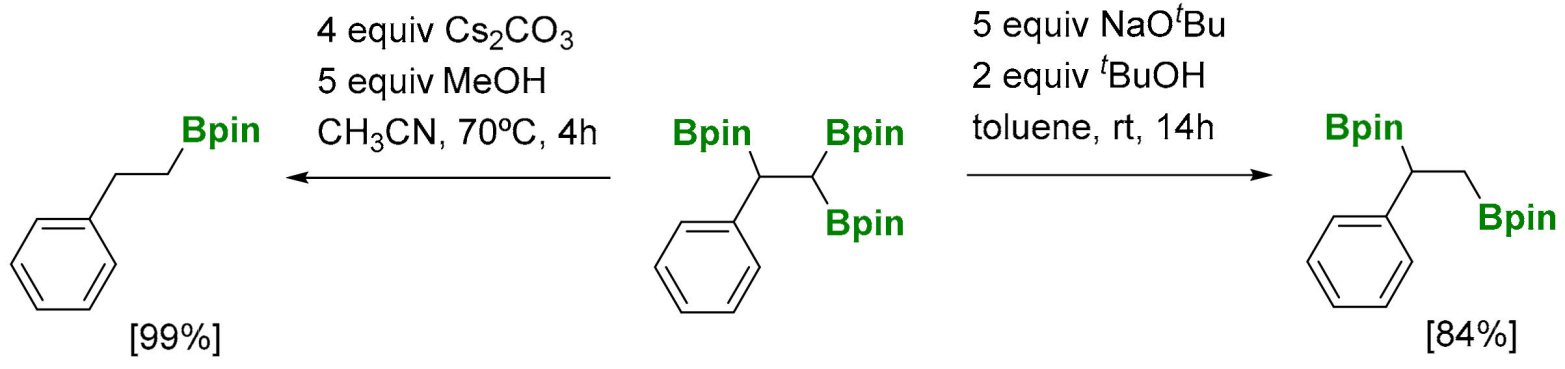
Selective deprotonation on the geminal diboryl moiety in 1,1,2-tri(boryl)alkanes.

The versatility of 1,1,2-tri(boryl)alkanes is based on the construction of new C-C and C-heteroatom bonds, since the three boronate moieties can be distinguished in a stepwise manner. Song and co-workers proved the selective deprotonation on the geminal diboryl moiety, when NaO^t^Bu is used as base (5 equiv) producing the 1,2-bis(boronate) product (Scheme 27) [29]. However, in the presence of Cs_2_CO_3_ (4 equiv) the 1,1,2-tri(boryl)alkane is converted into the linear alkyl monoboronate product (Scheme 27) [29].

**Scheme 28:**
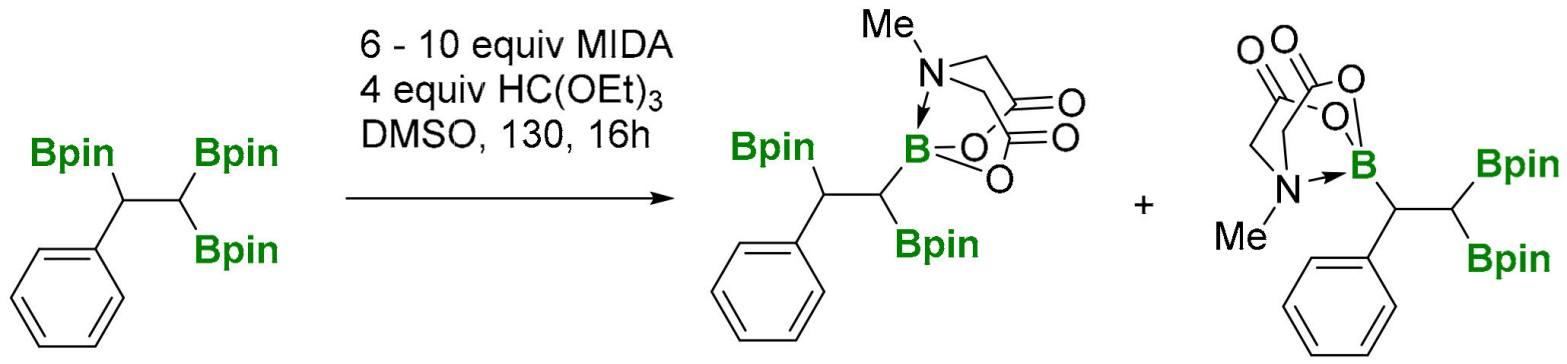
Transformation from 1,1,2-tri(boryl)alkane into mixed Bpin and B-MIDA triboronate compounds.

A simple transformation from 1,1,2-tri(boryl)alkane into mixed Bpin and B-MIDA triboronate compounds (MIDA=N-(methyliminodiacetic acid) has been developed by Sharma and co-workers [30]. However, the experiment showed that the treatment of 1,1,2-tri(pinacolboryl)alkane with MIDA provided a mixture (1:1 ratio by NMR) of the two mono-B(MIDA)-diBpin species, possessing the B(MIDA) group vicinal to Bpin or geminal to the Bpin moiety (Scheme 28). Unfortunately, the mixture resulted in being inseparable, using silica gel chromatography.

**Scheme 29:**
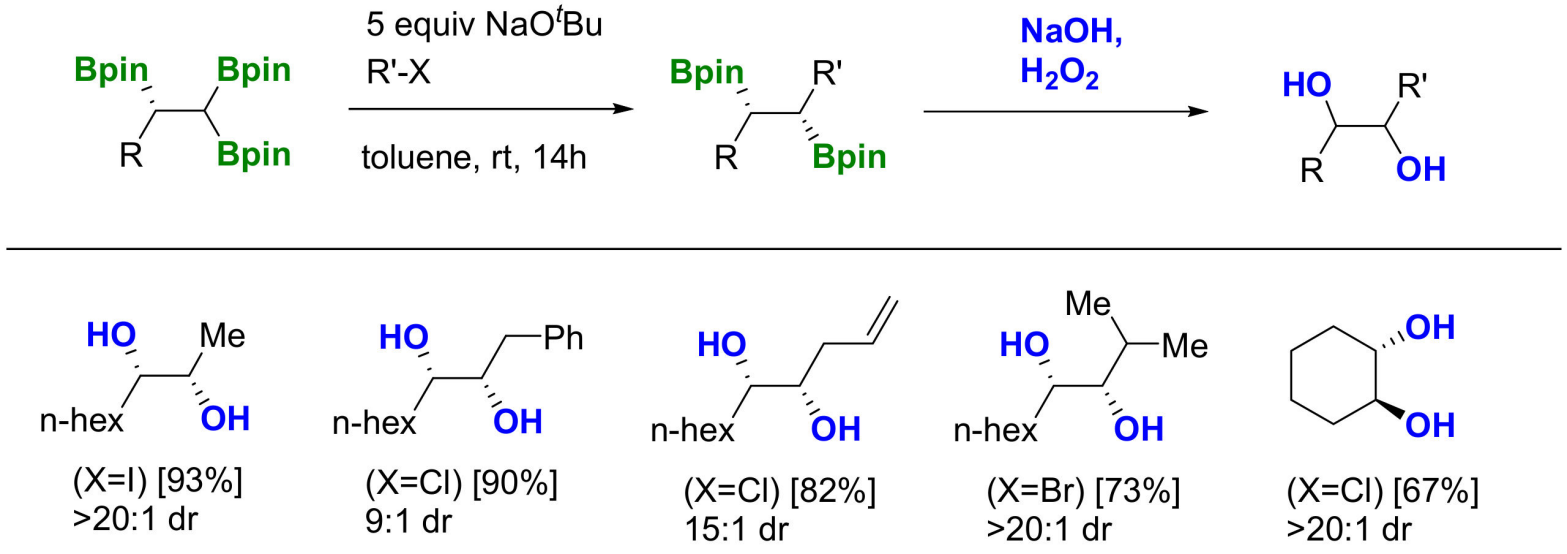
Deborylative alkylation with chiral 1,1,2-tris-(boronates).

Deborylative alkylation reactions with chiral 1,1,2-tris-(boronates) were performed in the group of Morken [28], and they found that five equivalents of NaO^t^Bu in toluene were efficient to form the new C-C bond from one of the geminal boryl moieties. The syn diastereoisomer was the predominant product, obtained as 1,2-diol upon oxidative workup. The reaction was applied for primary and seconary electrophiles (Scheme 29). The deborylative alkylation with alkenyl electrophiles represents a suitable method for the synthesis of internal vicinal bis(boronates), in the presence of terminal alkenes. When the substrate contains alkyl halide groups, the vinyl boronate diboration enables the construction of 1,1,2-tri(boryl)alkanes that may undergo intramolecular alkylation to form cyclic 1,2-bis(boronates). This method allows the construction of five, six and seven membered rings, which, after in situ oxidation, provides anti-1,2-diol (Scheme 29).

## 5. 1,1,2-Tri(boryl)alkenes

### 5.1. Synthetic Approaches

**Scheme 30:**
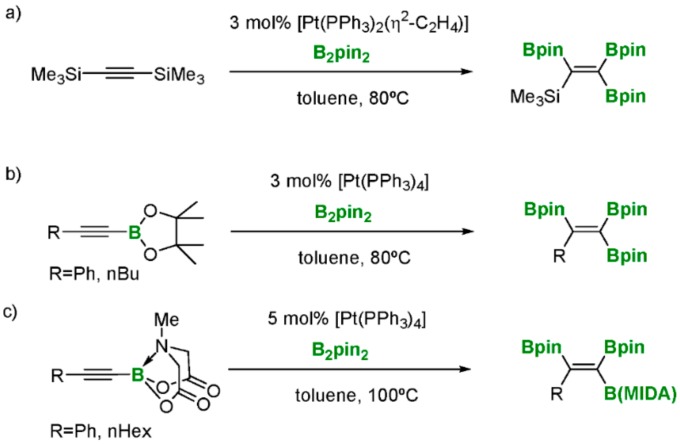
(**a**) Tandem desilylative borylation of bis(trimethylsilyl)acetylene and the subsequent Pt-catalyzed diboration; (**b**) Pt-catalyzed diboration of 1-alkynylboronates; (**c**) platinum-catalyzed diboration of phenylethynyl MIDA boronate.

The preparation of polyborated alkenes has originally been accomplished by Marder and co-workers, via the tandem desilylative borylation of bis(trimethylsilyl)acetylene and the subsequent diboration with B_2_pin_2_ in the presence of [Pt(PPh_3_)_2_(η^2^-C_2_H_4_)] as catalyst (Scheme 30a) [31]. Similarly, Srebnik and co-workers established the convenience of using [Pt(PPh_3_)_4_] as catalyst to diborate 1-alkynylboronates to easily obtain the expected 1,1,2-triborylalkenes (Scheme 30b) [32]. More extensively, Nishihara and co-workers launched a more general platinum catalyzed diboration of phenylethynyl MIDA boronate with B_2_pin_2_ that proceeds to yield 1,1,2-triboryl-2-phenylethene, with two different classes of the boron functionalities (Scheme 30c) [33]. This protocol uses the MIDA boronic group in the substrate, which is considered a masked group [34], towards strategic further selective C-B functionalization.

**Scheme 31:**
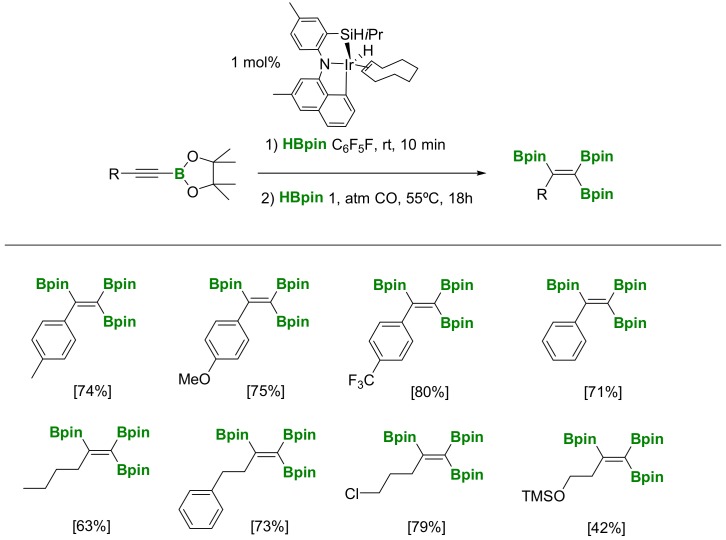
Dehydrogenative diboration with HBpin of alkynylboronates.

Moving from diboration to the hyroboration of alkynylboronates, Ozerov and co-workers discovered that pinacolborane (HBpin) can be efficiently added to terminal alkynes in the presence of iridium SiNC pincer complex, to efficiently prepare alkynylboronates. The following pathway involved the dehydrogenative diboration with HBpin and the same iridum complex under an atmosphere of CO (Scheme 31) [35]. The formation of 1,1,2-tri(boryl)alkanes through this protocol can be extended to a large number of substrates. However, the authors noted that the mechanism remains unclear, discarding the intermediacy of a hydroborated product or diboration with B_2_pin_2_.

**Scheme 32:**
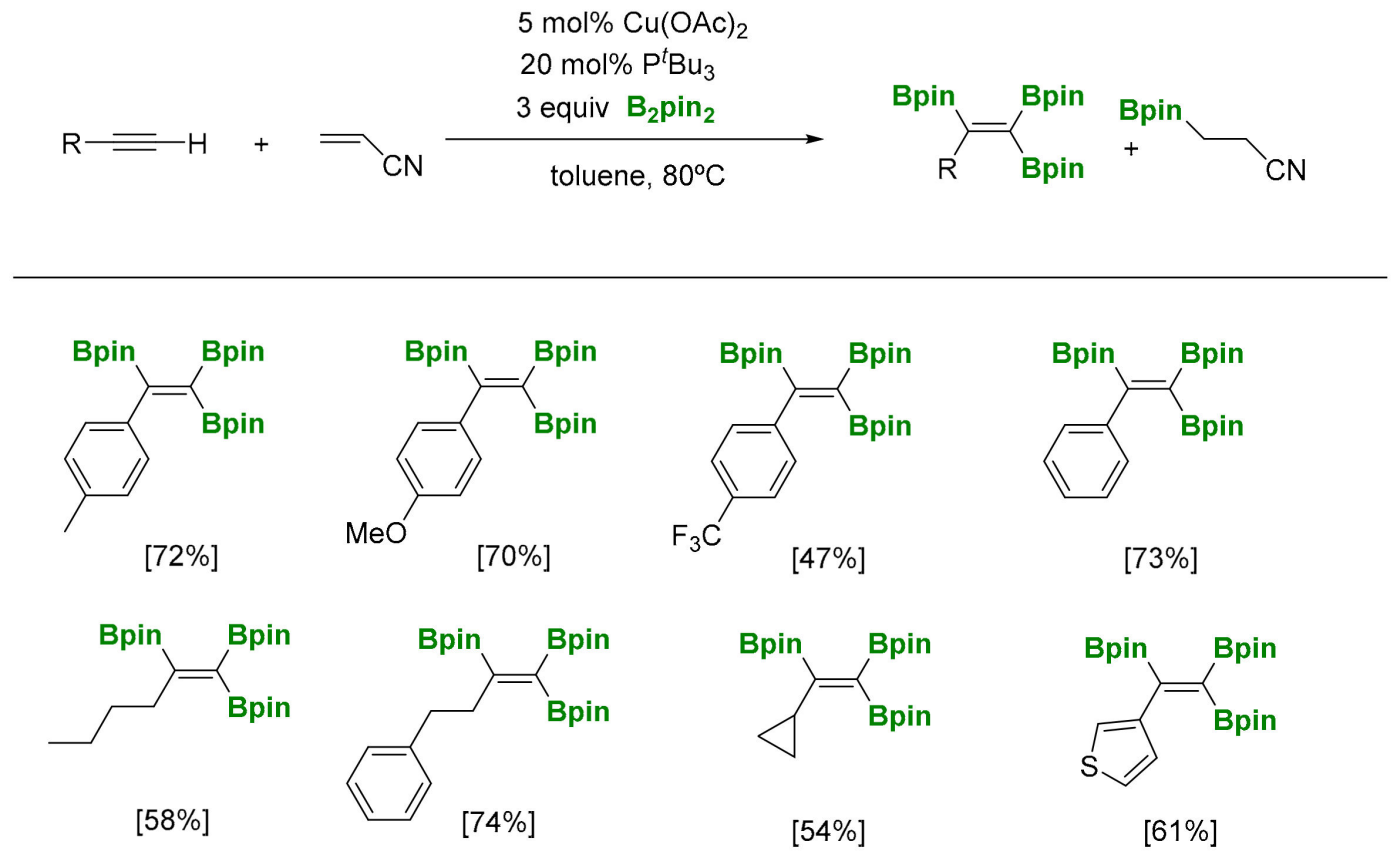
Copper catalyzed triboration of alkynes.

Marder and co-workers have developed a copper catalyzed triboration protocol for the synthesis of 1,1,2-triborylalkenes, using Cu(OAc)_2_ and P^n^Bu_3_ as suitable ligands, and with B_2_pin_2_ as a boron source (Scheme 32) [36]. The use of stoichiometric amounts of acrylonitrile as additive seems to be required as hydrogen (B-H) acceptor, in order to avoid by-products reactions. The reaction features mild reaction conditions, broad substrate scope and good functional group tolerance.

**Scheme 33:**
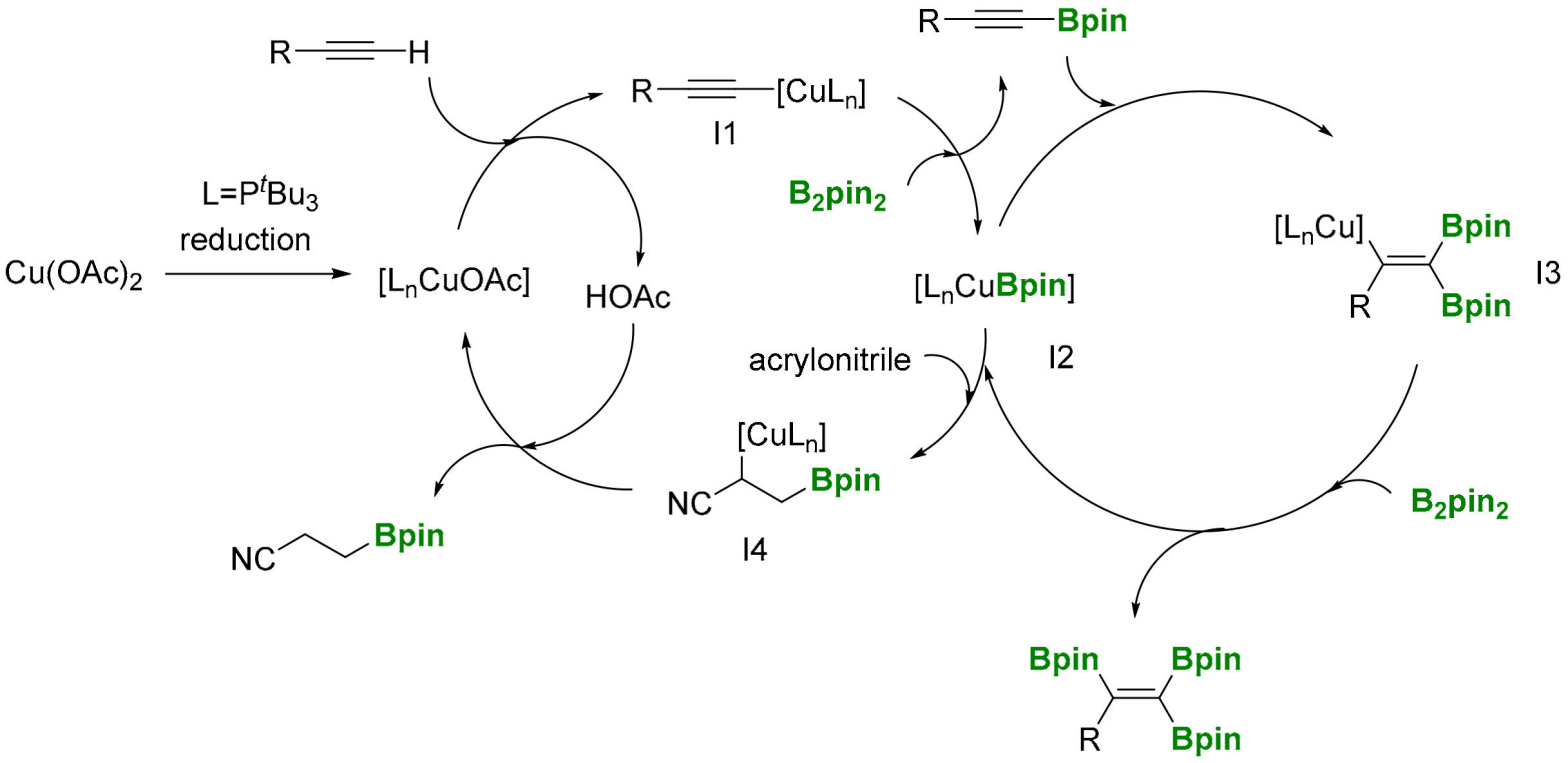
Mechanistic proposal of copper catalyzed triboration of alkynes.

The mechanism has been suggested on the basis of a coordination of terminal alkynes to [LnCuOAc], which is formed from Cu(OAc)_2_, the ligand and a reductive pathway (Scheme 33). Cu(OTf)_2_ or CuCl_2_ resulted inactive catalytic systems. Alternatively, the addition of KOAc to CuCl_2_ and CuCl reactivated the system, indicating that the ^−^OAc plays an important role in the reaction. The reactivity of [LnCuOAc] with the terminal alkyne provides the intermediate I1, which interacts with B_2_pin_2_ through σ-bond methatesis to afford the alkynylboronate and the Cu-Bpin species I2. Consequently, insertion of the alkynylboronate into Cu-Bpin generates the alkenylcopper I3 that eventually reacts with B_2_pin_2_ through σ-bond methatesis, to generate the desired 1,1,2-tri(boryl)alkene (Scheme 33). Acrylonirile is added, because its hydroboration is faster than that of alkynes, avoiding the alkyne hydroboration side reaction [36].

### 5.2. Synthetic Applications

**Scheme 34:**
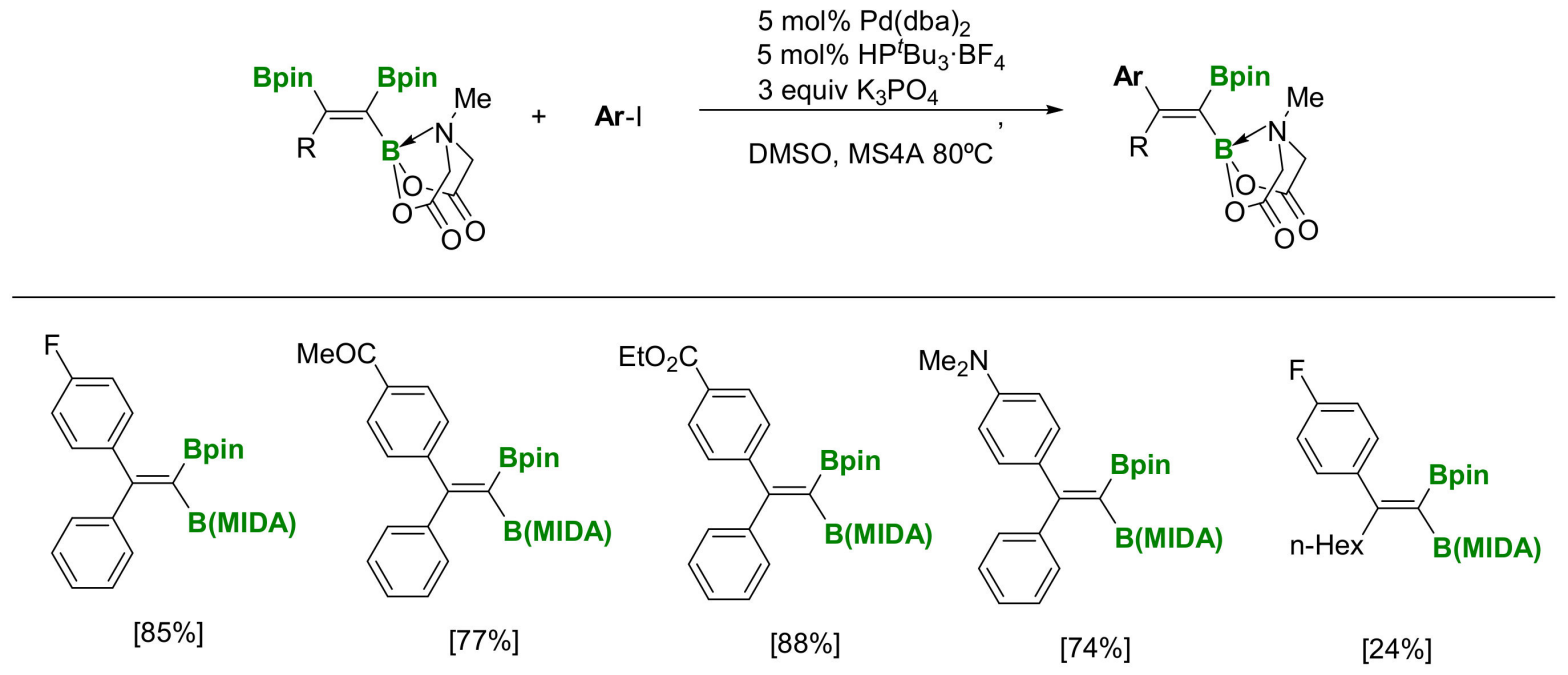
Selective palladium catalyzed arylation of 1,1,2-tri(boryl)alkenes.

The transformation of 1,1,2-tri(boryl)alkenes can be conducted by palladium-catalyzed arylation reaction with aryl iodides. Pd(dba)2 was found to be the best catalyst to give the arylated product and the new C-C is selectively formed in trans to the B(MIDA) moiety (Scheme 34). HP^t^Bu_3_BF_4_ was selected as the best ligand to modify the palladium complex in the presence of three equivalents of K_3_PO_4_ as the base [33]. The limitation of this protocol concerns the aryl halide used, since aryl iodide results in being very efficient for the cross-coupling reaction, and the aryl bromide and aryl chloride show a low or null reactivity (Scheme 34). Additionally, other electrophiles such as ethyl iodide or (E)-octenyl iodide were subjected to the Suzuki–Miyaura coupling reactions, but no desired products were formed. Despite the fact that some limitations were observed, this method represents the first example of obtaining gem-diborylated olefins with two different boryl groups via a chemoselective arylation. The chemoselectivity can be explained simply by a steric effect, because even the addition of two equivalents of aryl iodides did not provide the diarylated products [33].

**Scheme 35:**
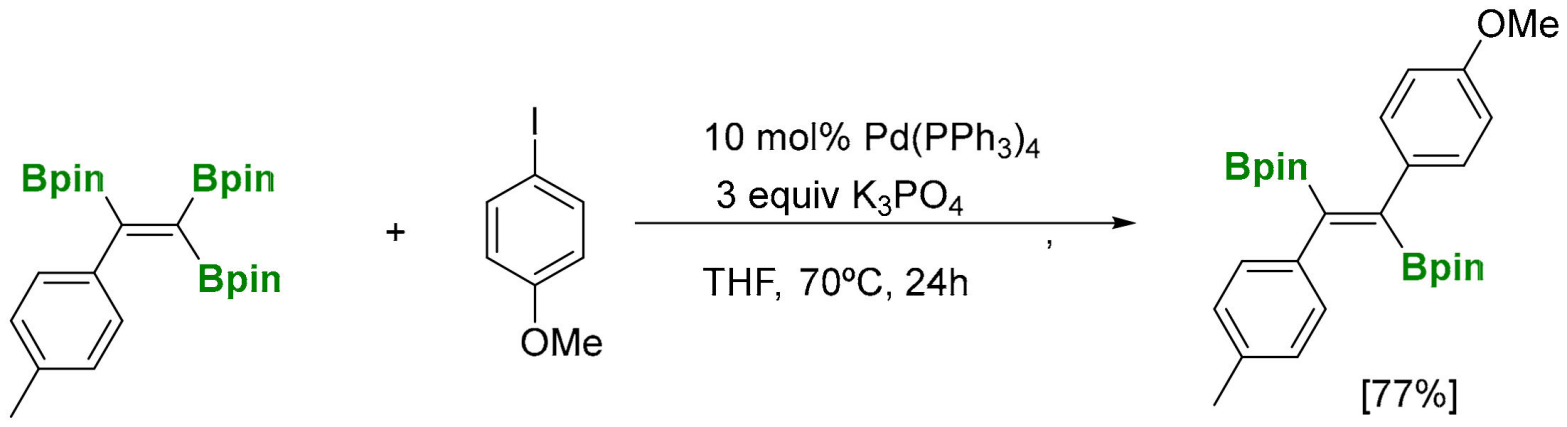
Selective palladium catalyzed arylation of 1,1,2-tri(boryl)alkenes.

The versatility of 1,1,2-tri(pinacolboryl)alkenes was also demonstrated, due to complementarily stereoselective Suzuki-Miyaura coupling reactions with aryl halides catalyzed by Pd(PPh_3_)_4_ in the presence of three equivalents of K_3_PO_4_ as the base (Scheme 35) [35]. The (E)-stereochemistry of the diborated product becomes one of the few examples described in the literature, and becomes a potential synthetic strategy to obtain trans-diaryldiborylalkenes in a simple way.

**Scheme 36:**
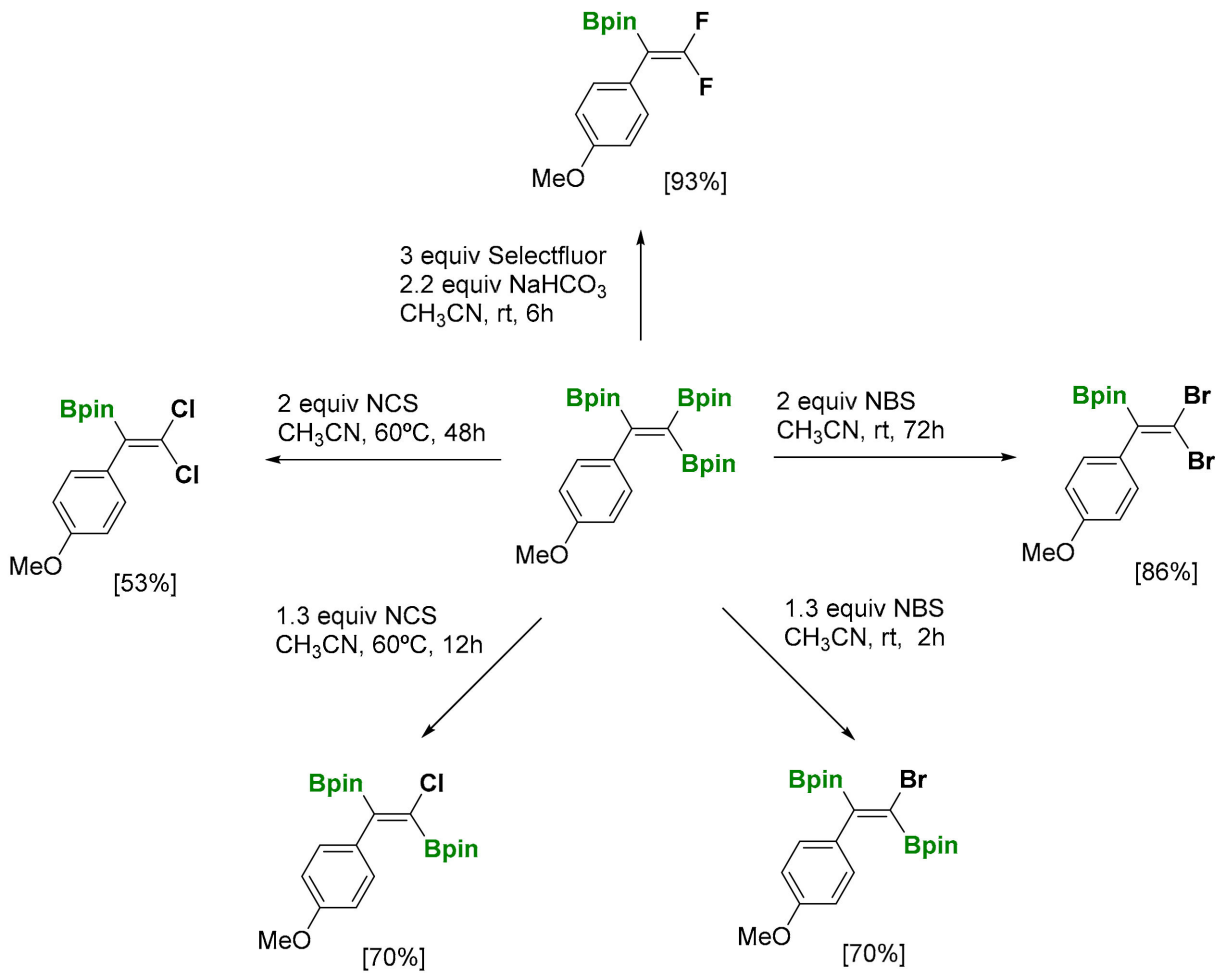
Selective functionalization of 1,1,2-tri(boryl)alkenes.

The utility of the 1,1,2-tri(pinacolboryl)alkenes has also explored different ways to be functionalized through difluorination (with Selectfluor) to afford gem-difluoroborylakenes (Scheme 36) [36]. Similarly, dibromination (with NBS) and dichlorination (with NCS) of 1,1,2-tri(pinacolboryl)alkenes facilitated the formation of gem-dihaloborylakenes (Scheme 36). The use of a lower amount of NBS or NCS, as well as lower reaction times, generated trans-stereoselectively towards the monohalo-diborylated alkene (Scheme 36).

## 6. Conclusions

The preparation of 1,1,1-tri(boryl)alkanes, 1,2,3-tri(boryl)alkanes, 1,1,2-tri(boryl)alkanes, as well as 1,1,2-tri(boryl)alkenes, appears to be a useful tool to synthesize organic compounds through selective C-B functionalization. The assistance of vicinal or geminal boryl units to the selective functionalization of C(sp^3^)-B or C(sp^2^-B) fragments becomes crucial. In part, this advantage is due to the enhanced α-borylcarbanions stability as intermediates in synthesis. Within the last decade, intensive efforts are devoted to prepare tri(boryl)alkanes and tri(boryl)alkenes, and significant applications have illustrated the powerful tool to construct molecules in a selective way. We can only expect that their interest will keep growing, attending to their real utility.
